# Human Pluripotent Stem Cell-Derived Striatal Interneurons: Differentiation and Maturation *In Vitro* and in the Rat Brain

**DOI:** 10.1016/j.stemcr.2018.12.014

**Published:** 2019-01-17

**Authors:** Zoe Noakes, Francesca Keefe, Claudia Tamburini, Claire M. Kelly, Maria Cruz Santos, Stephen B. Dunnett, Adam C. Errington, Meng Li

**Affiliations:** 1 Neuroscience and Mental Health Research Institute, School of Medicine, Cardiff University, Cardiff CF24 4HQ, UK; 2 School of Biosciences, Cardiff University, Cardiff CF10 3AX, UK

**Keywords:** human pluripotent stem cells, striatal interneurons, striatal development, medial ganglionic eminence, caudal ganglionic eminence, Huntington's disease, cell replacement therapy, differentiation

## Abstract

Striatal interneurons are born in the medial and caudal ganglionic eminences (MGE and CGE) and play an important role in human striatal function and dysfunction in Huntington's disease and dystonia. MGE/CGE-like neural progenitors have been generated from human pluripotent stem cells (hPSCs) for studying cortical interneuron development and cell therapy for epilepsy and other neurodevelopmental disorders. Here, we report the capacity of hPSC-derived MGE/CGE-like progenitors to differentiate into functional striatal interneurons. *In vitro*, these hPSC neuronal derivatives expressed cortical and striatal interneuron markers at the mRNA and protein level and displayed maturing electrophysiological properties. Following transplantation into neonatal rat striatum, progenitors differentiated into striatal interneuron subtypes and were consistently found in the nearby septum and hippocampus. These findings highlight the potential for hPSC-derived striatal interneurons as an invaluable tool in modeling striatal development and function *in vitro* or as a source of cells for regenerative medicine.

## Highlights

• hPSCs differentiate into cortical and striatal interneuron-like cells *in vitro*
• They present mature electrophysiological and morphological properties *in vitro*
• They express striatal interneuron subtype markers upon transplantation in rat brain
• hPSC-interneuron-like cells adopt region-specific morphologies *in vivo*

## Teaser

In this report, Noakes and colleagues highlight the importance of studying human striatal interneurons and demonstrate the presence of striatal interneuron markers in hPSCs differentiated toward MGE and CGE fate *in vitro*. HPSC-GABAergic neurons displayed functional intrinsic and network properties *in vitro*, and adopted striatal interneuron subtype fates *in vivo*, making them a suitable tool for further research.

## Introduction

The medial ganglionic eminence (MGE) and caudal ganglionic eminence (CGE) give rise to cortical, striatal and hippocampal interneurons, as well as globus pallidus projection neurons and cholinergic basal forebrain neurons. These cells are crucial for cortical and basal ganglia function; and their dysfunction has been implicated in diseases such as epilepsy, schizophrenia, autism, Huntington's disease (HD), and dystonia (Lewis, 2012, Powell et al., 2003, Reiner et al., 2013, Zikopoulos and Barbas, 2013). Given the correct patterning cues, human pluripotent stem cells (hPSCs) can differentiate into any cell type in the body, providing an excellent *in vitro* tool for the study of human neural development and function. On this topic, there has been much interest in using hPSCs to generate cortical or hippocampal GABAergic interneurons (Cambray et al., 2012, Kim et al., 2014, Maroof et al., 2010, Nicholas et al., 2013), and some efforts to produce cholinergic forebrain neurons (Bissonnette et al., 2011, Crompton et al., 2013). However, little attention has been paid to striatal interneurons despite their important role in dystonia and HD (Capetian et al., 2014, Reiner et al., 2013).

While interneurons comprise only 5%–10% of rodent striatal neurons, they make up more than 20% of primate striatal neurons, suggesting a more important role in primates than in rodents (Graveland and DiFiglia, 1985, Wu and Parent, 2000). The remaining striatal population are the projecting medium spiny neurons (MSNs), born in the adjacent lateral ganglionic eminence (LGE). Proof-of-principle studies showing functional improvement in HD animal models have used whole ganglionic eminence (WGE) comprising both LGE and MGE fetal tissue (Kendall et al., 1998, Palfi et al., 1998). Interneurons will likely be essential for modeling striatal function with hPSCs, and may help transplanted hPSC-derived LGE-like cells to differentiate into MSNs and integrate *in vivo* for the treatment of HD.

Striatal interneurons fall into four main subtypes with distinct molecular and functional characteristics. Parvalbumin (PV)- and somatostatin (SST)-expressing GABAergic interneurons and choline acetyltransferase (ChAT)-expressing cholinergic interneurons are born in the MGE marked by transcription factor NKX2.1. Most calretinin (CR) interneurons arise from COUP-TFII-expressing progenitors in the CGE (Butt et al., 2005, Marin et al., 2000). The only known molecular profile that reliably distinguishes MGE-derived striatal and cortical interneurons *in vivo* is co-expression of NKX2.1 and LHX6. While cortical interneurons switch off expression of NKX2.1 on post-mitotic upregulation of LHX6, striatal interneurons maintain expression of both transcription factors into adulthood (Nobrega-Pereira et al., 2008).

The common developmental origin of cortical and striatal interneurons indicates that differentiating hPSCs toward cortical interneurons should also produce striatal interneurons. Here we show the production of GABAergic interneurons of each subtype *in vitro* from human embryonic stem cells (hESCs), which demonstrated maturing electrophysiological properties. Upon transplantation into the neonatal rat striatum, hESC-derived neural progenitors differentiated into striatal CR and cholinergic interneurons and showed region-specific morphology depending on where they settled.

## Results

### HESC-Derived MGE- and CGE-like Progenitors Give Rise to Striatal Interneuron-like Cells *In Vitro*

**Figure 1:**
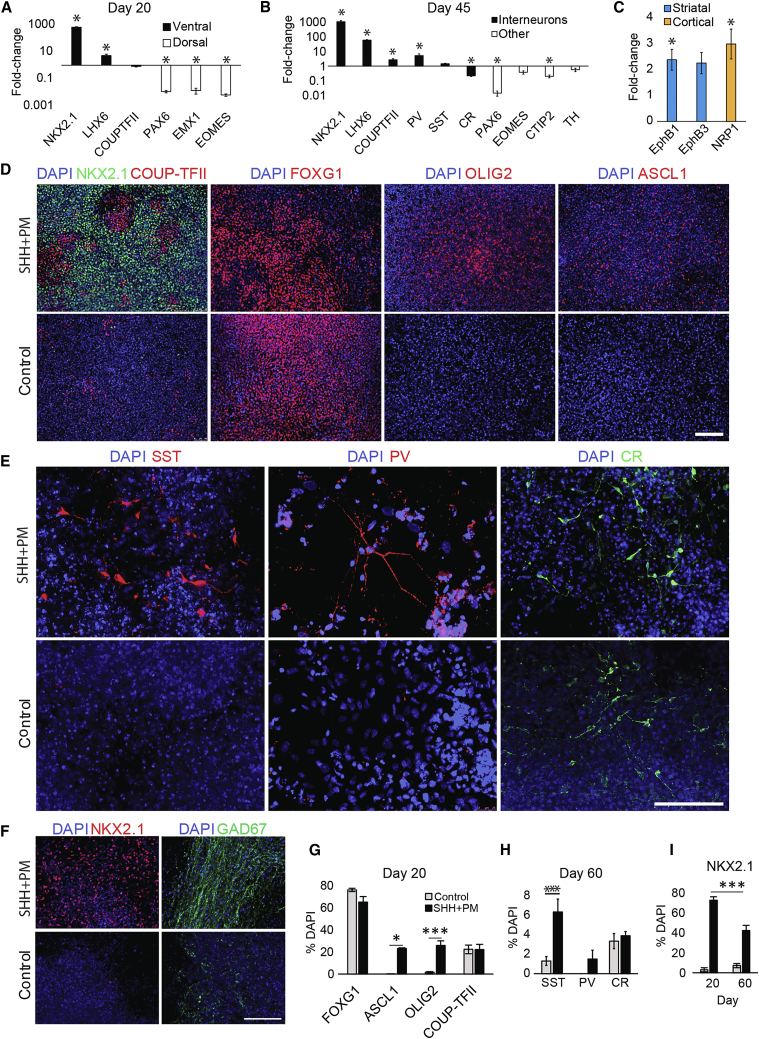
Differentiation of hESCs into Striatal and Cortical Interneurons *In Vitro* (A–C) qPCR data presented as gene expression fold-change of SHH-treated samples relative to untreated controls on days 20 (A) and 45 (B) on a logarithmic y axis. Day 45 samples were also analyzed for their expression of cortical and striatal interneuron-specific markers (C). Data are presented as mean fold-change ± SEM from three independent experiments performed in H7 cells. ^∗^p < 0.05, two-sample t test with equal variance not assumed. (D–F) Representative immunocytochemistry images of H7 control and SHH-treated cultures at days 20 (D) and 60 (E and F). Scale bars, 100 μm. (G–I) Images were counted for MGE and CGE progenitor markers (G and I) and interneuron subtype markers (H and I). Data presented are mean ± SEM from independent experiments performed in H7 (n = 3), H7-tauGFP (n = 2), H9 (n = 1), and iCas9 (n = 1) for day 20 and H7 (n = 3) and H7-tauGFP (n = 2) for day 60. ^∗^p < 0.05, ^∗∗∗^p < 0.001, one-way ANOVA (COUP-TFII, SST, CR), Kruskal-Wallis test (FOXG1, ASCL1, OLIG2, PV), and two-way ANOVA with *post hoc* Bonferroni (NKX2.1).

hESCs seeded in a monolayer underwent neural induction by dual-Smad inhibition, with rostral fate facilitated by WNT signaling inhibitor, XAV939. This was followed by combinatorial treatment of sonic hedgehog (SHH) and purmorphamine to induce ventral forebrain identity. We performed qPCR analyses at days 20 (D20) and 45 (D45) of differentiation. At D20, SHH-treated cultures showed a marked increase of MGE markers *NKX2.1* and *LHX6*, and decrease of dorsal forebrain markers *PAX6*, *EMX1*, and *EOMES* compared with untreated controls (Figure 1A; Table S1). Little change was observed for CGE marker *COUP-TFII* between conditions. At D45, *NKX2.1*, and *LHX6* expression remained highly elevated in SHH-treated cultures, and COUP-TFII expression significantly increased to nearly three times that of control cultures (Figure 1B). Expression of interneuron subtype marker *PV* was five times higher in SHH-treated samples, but the marginal increase in *SST* expression was not significant. In contrast, *CR* expression was significantly lower in SHH-treated cultures, falling to 20% of that of controls. Expression of genes specific to regions other than the MGE or CGE were reduced or similar in SHH-treated cultures compared with controls. The transcript levels of MGE and post-mitotic interneuron marker genes in SHH-treated cultures were higher at D45 than those of D20; and were either similar or higher than those of 15-gestational-week human fetal MGE, apart from *LHX6* and *PV* (Figure S1A).

Striatal and cortical interneurons express distinct guidance molecules such that they respond differently to migratory cues and settle in the striatum or cortex (Nobrega-Pereira et al., 2008, Villar-Cervino et al., 2015). *EPHB1* and *EPHB3*––expressed by striatal interneurons––and cortical interneuron marker *NRP1*, showed increased mRNA levels in SHH-treated cultures compared with controls (Figure 1C; Table S1). This shows that SHH treatment led to increased transcription of MGE- and CGE-derived interneuron genes, and notably elevated levels of mRNA specific to both striatal and cortical interneuron guidance molecules.

Preferential induction of MGE fate in SHH-treated cultures was confirmed at the protein level by immunostaining of interneuron and progenitor markers. Most cells in D20 SHH-treated cultures expressed NKX2.1, and around a quarter of cells expressed ASCL1 and OLIG2 (Figures 1D, 1G, and 1I; Table S2). COUP-TFII expression was around 22% in both conditions, but in SHH-treated cultures its expression pattern appeared opposite to that of NKX2.1, with few cells expressing both proteins (Figure 1D). No statistical difference was found in the number of cells expressing FOXG1, a marker for all forebrain neural progenitors (Figures 1D and 1G). At D60, there was a higher proportion of SST^+^ cells in those treated with SHH (SHH 6.3% ± 1.3%, Ctrl 1.3% ± 0.5%, p < 0.001) (Figures 1E and 1H). PV^+^ cells were detected in three out of six experiments in SHH-treated cultures (1.5% ± 0.9%) but never observed in control cultures. CR was expressed in 3%–4% of cells in both conditions. SHH-treated cultures maintained a greater proportion of NKX2.1^+^ cells at D60, but presented a significant drop of 30% compared with D20 (Figures 1F and 1I). GAD67^+^ cells were widely observed in SHH-treated cultures, in visibly greater numbers than in controls (Figure 1F). Comparable numbers of MAP2^+^ and NeuN^+^ neurons were detected in the control and SHH-treated cultures (Figure S1B), suggesting that SHH treatment did not affect overall neuronal production. Together, these results confirm the generation of neurons resembling cortical and striatal GABAergic interneurons of different subtypes.

### HESC-Derived Interneurons Develop Mature Electrophysiological and Morphological Properties *In Vitro*

**Figure 2:**
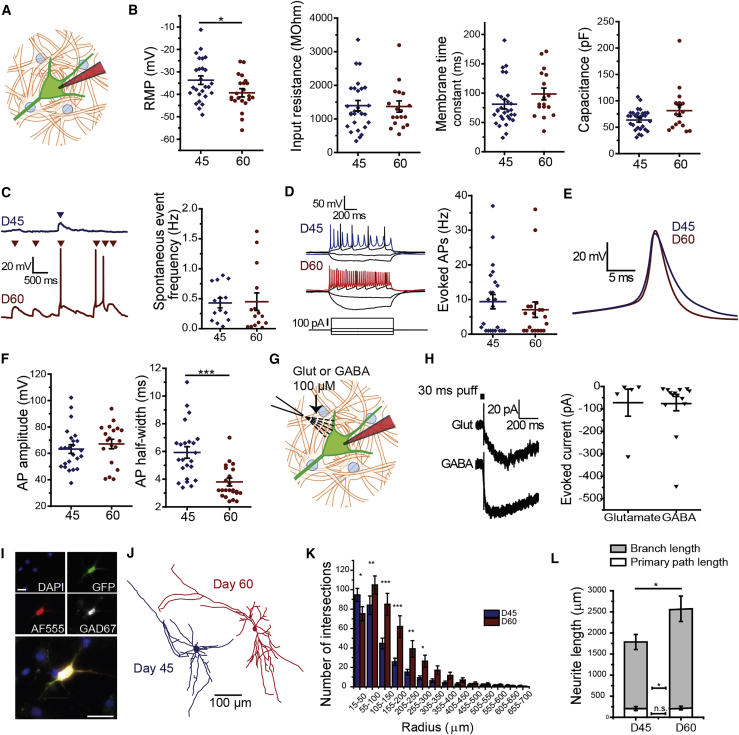
hESC-Derived MGE/CGE-like Progenitors Become Functional GABAergic Neurons (A) Schematic of whole-cell patch clamp of H7-tauGFP^+^ neurons co-cultured with primary mouse astrocytes. (B) Basic membrane properties at days 45 (n = 25) and 60 (n = 16–19). RMP, resting membrane potential. (C) Representative traces of spontaneous activity (left). Post-synaptic potentials and action potentials were counted over 2 min (right: D45, n = 14; D60, n = 14). (D) Representative traces of evoked activity from current injection steps (left). The maximum number of evoked spikes was quantified in each cell (right: D45, n = 23; D60, n = 19). (E) Overlaid averaged traces of all D45 (blue, n = 15) and D60 (red, n = 16) first evoked spikes. (F) Amplitude and half-width of first evoked spikes. (G) Schematic illustrating second pipette for focal application of glutamate or GABA onto patched cell. (H) Representative traces showing glutamate- (Glut, 100 μM) and GABA (100 μM)-evoked currents (left) and their quantification (right: Glut, n = 5; GABA, n = 14). (I) *Post hoc* immunocytochemistry of AF555-filled neurons to confirm GAD67 (white) expression. Scale bars, 15 μm. (J) AF555-filled neurons were imaged and traced *post hoc* in Neurolucida. (K) Quantification of Sholl analysis intersections compared by two-way ANOVA with *post hoc* Bonferroni correction. (L) Total neurite length was divided into primary path length (white) and branch length (gray) (D45, n = 15; D60, n = 18). n represents number of cells from three independent experiments and all data plots show mean ± SEM of the cells recorded. All statistical analyses except for (K) were done by two-sample t test. ^∗^p < 0.05, ^∗∗^p < 0.01, ^∗∗∗^p < 0.001.

We next assessed the functional maturation of hESC-derived neurons using whole-cell patch-clamp electrophysiology (Figure 2A). HESC-neural derivatives were co-cultured with mouse astrocytes from passage 2 to promote neuronal maturation. An H7 derivative line that constitutively expresses a cytoplasmic TauGFP fusion protein was used for easier identification of human neurons (Pratt et al., 2000). First, using current clamp we measured intrinsic membrane properties at differentiation D45 and D60. The mean resting membrane potential decreased significantly, indicating functional maturation of the cells (Figure 2B; Table S3). However, there was no significant change in input resistance, membrane time constant, or capacitance of the cells.

To assess the intrinsic excitability of our hESC-derived neurons and their ability to form functional synapses, we calculated the frequency of spontaneous events (action potentials and post-synaptic potentials) and evoked spikes (Figures 2C and 2D; Table S3). Most cells displayed spontaneous activity and fired evoked trains of action potentials, but there was no significant change in the mean frequency of either over time. However, comparison of individual action potential kinetics revealed a 35% reduction in mean spike half-width––indicating an increase in the number of voltage-gated ion channels in the cell membrane––despite only a modest increase in mean spike amplitude (Figure 2F).

Striatal neurons are subject to both glutamatergic input from the cortex and thalamus, and GABAergic input from local connections. We tested the effects of focally applied glutamate and GABA in our D60 neurons using voltage clamp (Figure 2G). Held at −45 mV, glutamate evoked inward currents in three out of five cells (Figure 2H; Table S3). At a holding potential of −70 mV, all cells responded to GABA with an inward current driven primarily by GABA_A_ receptors, verified by blocking with picrotoxin.

Finally, we investigated the molecular and morphological development of the hESC-derived neurons. *Post hoc* immunostaining confirmed that 60.6% of GFP^+^ cells in the cultures expressed GAD67 (Figure 2I). During patch-clamp recordings, neurons were filled with Alexa Fluor 555 (100 μM) and imaged *in situ* for reconstruction using Neurolucida 360 (Figure 2J). Sholl analysis revealed significantly increased neurite complexity at D60, shown by a greater number of intersections up to 300 μm (Figure 2K). The significant difference in total neurite length was driven entirely by the growth of branches, while mean primary neurite length remained constant (Figure 2L; Table S3). Together, these data show that hESCs can differentiate into functional, morphologically complex neurons expressing relevant neurotransmitter receptors *in vitro*, making them a suitable platform with which to study neural functional development.

### Transplanted hESC-Derived MGE/CGE-like Progenitors Give Rise to Striatal Interneuron-like Cells in Rat Striatum

**Figure 3:**
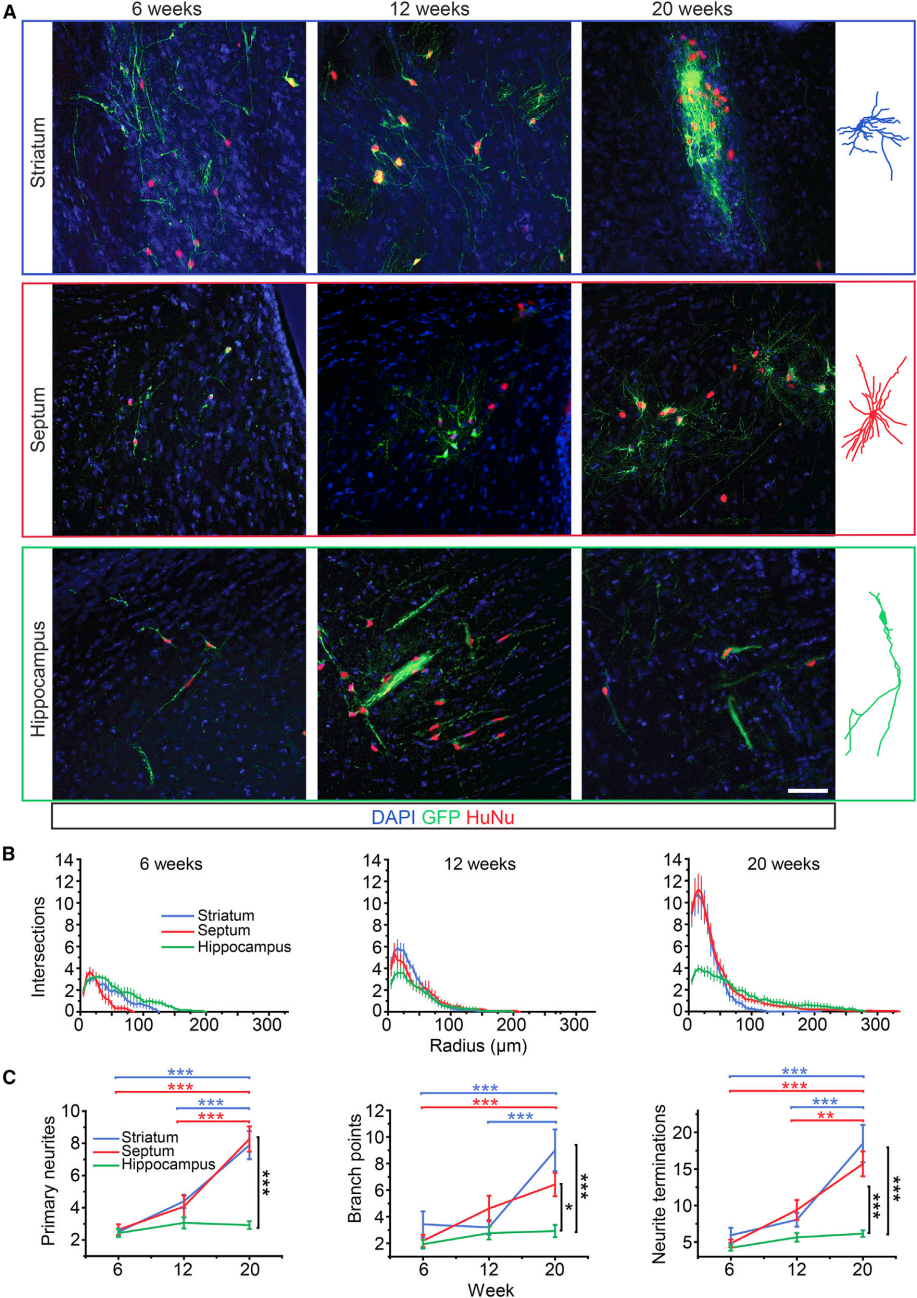
Transplanted MGE/CGE-like Progenitors Adopt Region-Specific Morphologies (A) Representative immunohistochemistry images of H7-tauGFP^+^ (green) hESC-derived progenitors and neurons stained for HuNu (red) and counterstained with DAPI (blue) at 6, 12, and 20 weeks post-transplantation, in the striatum, septum, and hippocampus of rats. Visible GFP^+^ cells were traced using Neurolucida and representative examples are shown on the right. Scale bar, 50 μm. (B) Sholl profiles comparing cells in each brain region at 6, 12, and 20 weeks. Data presented are mean number of intersections per shell ± SEM of n = 9–26 cells. (C) The number of primary neurites, branch points, and terminations were compared by two-way ANOVA with *post hoc* Bonferroni. Horizontal bars show significant differences across time points color-coded to their respective brain regions (blue, striatum; red, septum; green, hippocampus) and black vertical bars show significant differences between brain regions at 20 weeks. ^∗^p < 0.05, ^∗∗^p < 0.01, ^∗∗∗^p < 0.001.

We next explored the potential of the cells to adopt a striatal interneuron phenotype *in vivo*. D20 MGE/CGE-like progenitors, derived from the GFP^+^ H7 line, were transplanted into the right striatum of rat pups immune-suppressed with cyclosporine A. At 6 weeks post-transplantation (WPT), GFP^+^ cells were found in the striatum, septum, and hippocampus of four out of six recipients, and were confirmed to always co-label for HuNu despite some variation in GFP brightness (Figures 3A and S2). Neuronal morphology complexified over time, with a significant increase in the number of primary neurites by 20 WPT in the striatum and septum (Figures 3B and 3C). Cells in the striatum and septum also developed a significantly greater number of branch points and neurite terminations, indicating a difference in morphological development between cells that settled in the different regions (Figure 3C). These observations suggest that environmental cues had an impact on neuronal morphology, and that transplanted cells integrated themselves structurally within the host brain.

**Figure 4:**
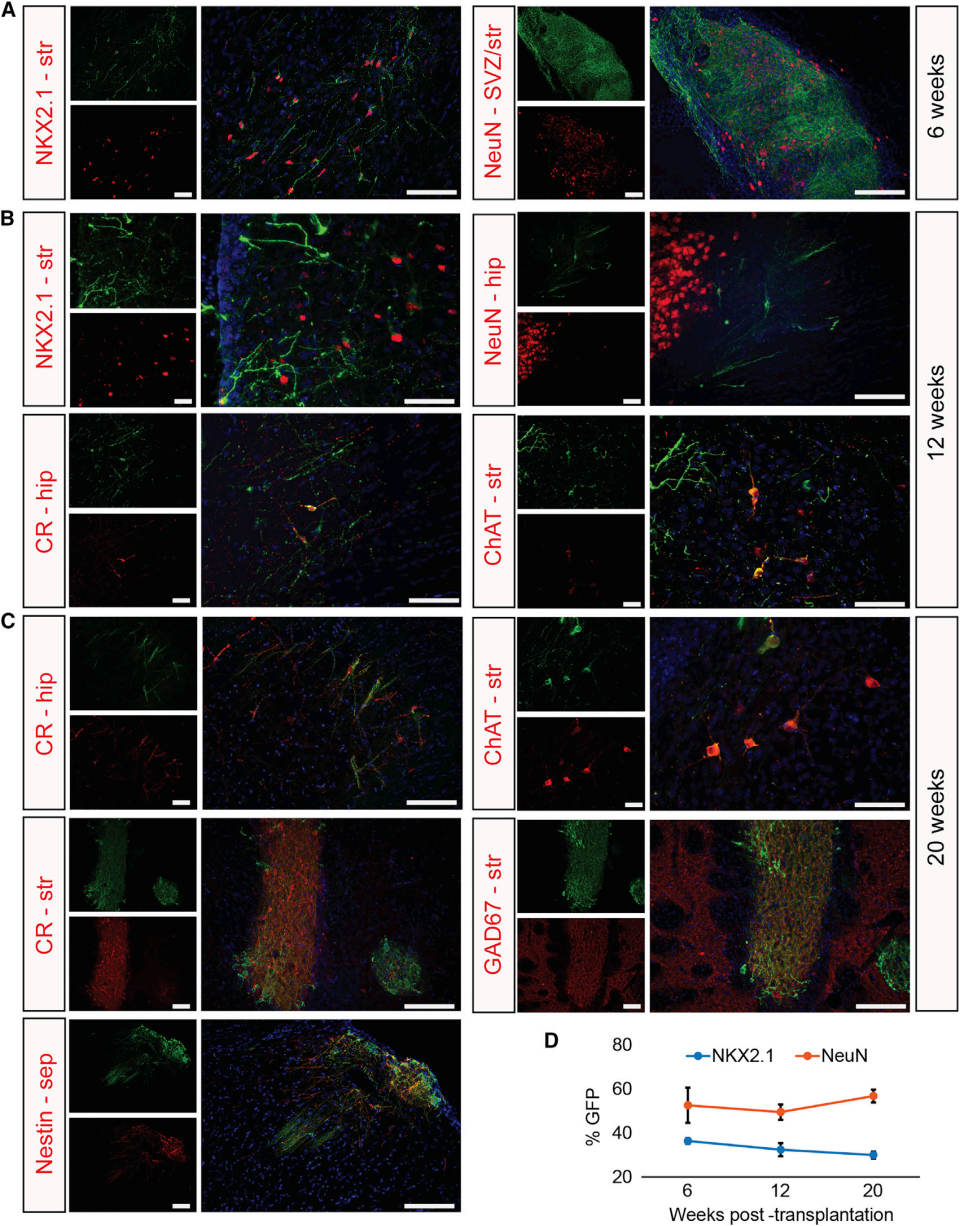
Transplanted MGE/CGE-like Progenitors Differentiate into Striatal Interneurons (A–C) Representative immunohistochemistry images of brain sections from 6 (A), 12 (B), and 20 (C) weeks post-transplantation, with GFP^+^ (green) transplanted cells and DAPI (blue). (D) NKX2.1 (blue: 6 weeks, n = 6; 12 weeks, n = 5; 20 weeks, n = 3) and NeuN (orange: 6 weeks, n = 5; 12 weeks, n = 3; 20 weeks, n = 3) were counted as a percentage of HuNu^+^ and GFP^+^ cells, respectively. Two-way ANOVA reported no significant differences. Str, striatum; SVZ, subventricular zone; hip, hippocampus; sep, septum. Scale bars, 100 μm (A–C, top two panels), 250 μm (C, bottom three panels).

Immunostaining of the grafted brains revealed that around half of surviving cells had differentiated into post-mitotic neurons by 6 WPT, demonstrated by NeuN staining, which did not change at 12 or 20 WPT (Figures 4 and S2). Consistent with this, we observed Nestin staining in more than 40% of GFP^+^ cells at 20 WPT, suggesting protracted neuronal differentiation *in vivo* (Figure 4C). NKX2.1 expression remained stable in a third of GFP^+^ cells over time, and there was no difference across the brain regions in which cells settled (Figure 4). Again this indicates that many cells had differentiated by 6 WPT, and either that a pool of cells remained NKX2.1^+^ progenitors, or that they maintained its expression––as would mature striatal interneurons in normal development (Nobrega-Pereira et al., 2008) We observed synaptophysin^+^ puncta confirming the presence of synapses on transplanted cells, although we cannot conclude whether these were from host innervation (Figure S2).

Looking at interneuron subtype-specific markers, CR was widely expressed throughout the grafts at 12 and 20 WPT, both in the striatal graft cores and in morphologically mature neurons in the striatum, septum, and hippocampus (Figures 4B and 4C). Neither PV nor SST expression were observed in any GFP^+^ cells, unlike sister cultures that matured *in vitro* (Figure 1). However, ChAT––a cholinergic interneuron marker not observed *in vitro* but normally present in the striatum––was expressed in a small number of striatal GFP^+^ cells at 12 and 20 WPT (Figures 4B and 4C). GAD67 was highly expressed throughout the grafted cells, corroborating the GABAergic identity of the CR^+^ neurons (Figure 4C). Finally, we ruled out any unwanted effects of cyclosporine A on the differentiation and survival of the cells, by treating *in vitro* cultures for up to 7 weeks (Figure S3). These results show that hESC-derived MGE/CGE-like progenitors have the capacity to differentiate into cells resembling striatal interneurons *in vivo*, as well as adopting septal and hippocampal interneuron-like fates having settled in these regions.

## Discussion

In this study, we have shown that hESCs can differentiate into striatal interneurons. *In vitro*, MGE/CGE-like progenitors gave rise to SST^+^, PV^+^, and CR^+^ neurons, which normally populate the cortex, striatum, and hippocampus. After transplantation into the neonatal rat striatum, they produced striatal interneuron-like cells expressing CR and ChAT. Consistent with this, both *EPHB1/3* (striatal) and *NRP1* (cortical) transcripts were present in our cultures (Marin et al., 2001, Villar-Cervino et al., 2015). Thus, striatal transplantation of hESC-derived MGE or CGE progenitors results in a bias toward striatal interneuron fate, rather than cortical interneuron fate following cortical transplantation.

The discrepancies between interneuron subtypes obtained in culture and in the rat striatum are intriguing. Neuronal differentiation *in vivo* was delayed in comparison with *in vitro* cultures––a phenomenon we have observed previously in hESC-MSN transplantation (Arber et al., 2015). CR interneurons appear relatively early in human cortical development, around gestational week 6, whereas SST and PV interneurons appear only sparsely around gestational week 20, perhaps explaining their total absence from the grafted cells in this study (Maroof et al., 2013, Nicholas et al., 2013, Zecevic et al., 2011). In the human striatum, there are three times more CR interneurons than PV or SST (Wu and Parent, 2000). Studies in mice and humans have shown that not all CR interneurons are derived from the CGE, and that most striatal CR interneurons are MGE-derived (Marin et al., 2000, Wang et al., 2014). Furthermore, research has shown that the host brain region is able to alter the fate of transplanted cells to more closely resemble its own (Quattrocolo et al., 2017). It is therefore reasonable to hypothesize that local cues favored either the survival of fate-committed CR and ChAT neurons, or their differentiation from the surviving progenitors. Future work could be designed to address this question by transplanting MGE/CGE-like progenitors to different brain regions, or at a later time point when they might be more fate-committed.

Neurotherapeutic strategies for HD may require the inclusion of striatal interneurons, as proof-of-concept has been provided by transplanting WGE––containing both MGE and LGE––into rodents, monkeys, and human patients (Barker et al., 2013, Kendall et al., 1998, Lelos et al., 2016, Palfi et al., 1998). Experimental HD therapy using hESC-derived MSNs also suggests a potential role for interneurons (Ma et al., 2012, Wu et al., 2018). These hESC-MSN preparations likely contain interneurons as they are induced by SHH using paradigms similar to interneuron induction (Ma et al., 2012). Therefore, optimizing striatal interneuron differentiation, maintenance, and transplantation will be vital for future *in vitro* and *in vivo* studies into striatal development, function and repair.

This article presents an original application for hPSC-derived MGE/CGE-like interneurons. We have highlighted a need for better understanding of the mechanisms behind the fate determination of forebrain interneurons and their role within the striatum. The stark differences in striatal interneuron numbers between humans and rodents indicates that we should be looking for such answers in human cells.

## Experimental Procedures

### HESC Culture and Differentiation

Three independent hESC lines (H7, H9, and iCas9-HUES9) and an H7 derivative line (H7-tauGFP) were used in this study. Routine hESC culture and interneuron differentiation methods, and the derivation of the H7-tauGFP^+^ cells are provided in Supplemental Experimental Procedures.

### Transplantation

All animal work was done in compliance with the European Directive 2010/63/EU on the protection of animals used for scientific purposes. All surgical procedures and injection coordinates are described in Supplemental Experimental Procedures.

### Statistical Analyses

All data were collected from at least three independent experiments, and are presented as mean ± SEM. Data were tested for normality with the Shapiro-Wilk test, and for equal variance with the Levene test, before performing statistical analyses by unpaired t test, ANOVA or non-parametric alternatives as stated in the figure legends. *Post hoc* Bonferroni test was applied following ANOVA to correct for multiple comparisons. All statistical tests were performed in Origin (OriginLab) or SPSS (IBM).

## Author Contributions

*In vitro* hESC culture, differentiation, qPCR and immunostaining was carried out by Z.N., F.K., C.T., and M.C. Electrophysiology was performed by Z.N. *In vivo* work was conducted by Z.N. and C.M.K., and Z.N. did perfusions, tissue sectioning and immunohistochemistry. All imaging, cell counting and *post hoc* analyses were done by Z.N. or F.K. S.B.D., A.C.E., and M.L. provided guidance and conceptual support. All authors edited the manuscript.

## Supplemental Information

Document S1. Supplemental Experimental Procedures, Figures S1–S3, and Tables S1–S3

Document S2. Article plus Supplemental Information
