# Supplementary material for: Human Pluripotent Stem Cell-Derived Striatal Interneurons: Differentiation and Maturation In Vitro and in the Rat Brain
Source: Stem Cell Reports. 2019 Jan 17;12(2):191–200. doi: 10.1016/j.stemcr.2018.12.014 (PMC6373547; doi:10.1016/j.stemcr.2018.12.014)
Supplement: Document S1. Supplemental Experimental Procedures, Figures S1–S3, and Tables S1–S3 [file mmc1.pdf]

**Stem Cell Reports, Volume *12***

## Supplemental Information

# Human Pluripotent Stem Cell-Derived Striatal Interneurons: Differentiation and Maturation *In Vitro* and in the Rat Brain

**Zoe Noakes, Francesca Keefe, Claudia Tamburini, Claire M. Kelly, Maria Cruz Santos, Stephen B. Dunnett, Adam C. Errington, and Meng Li**

Supplemental Figures

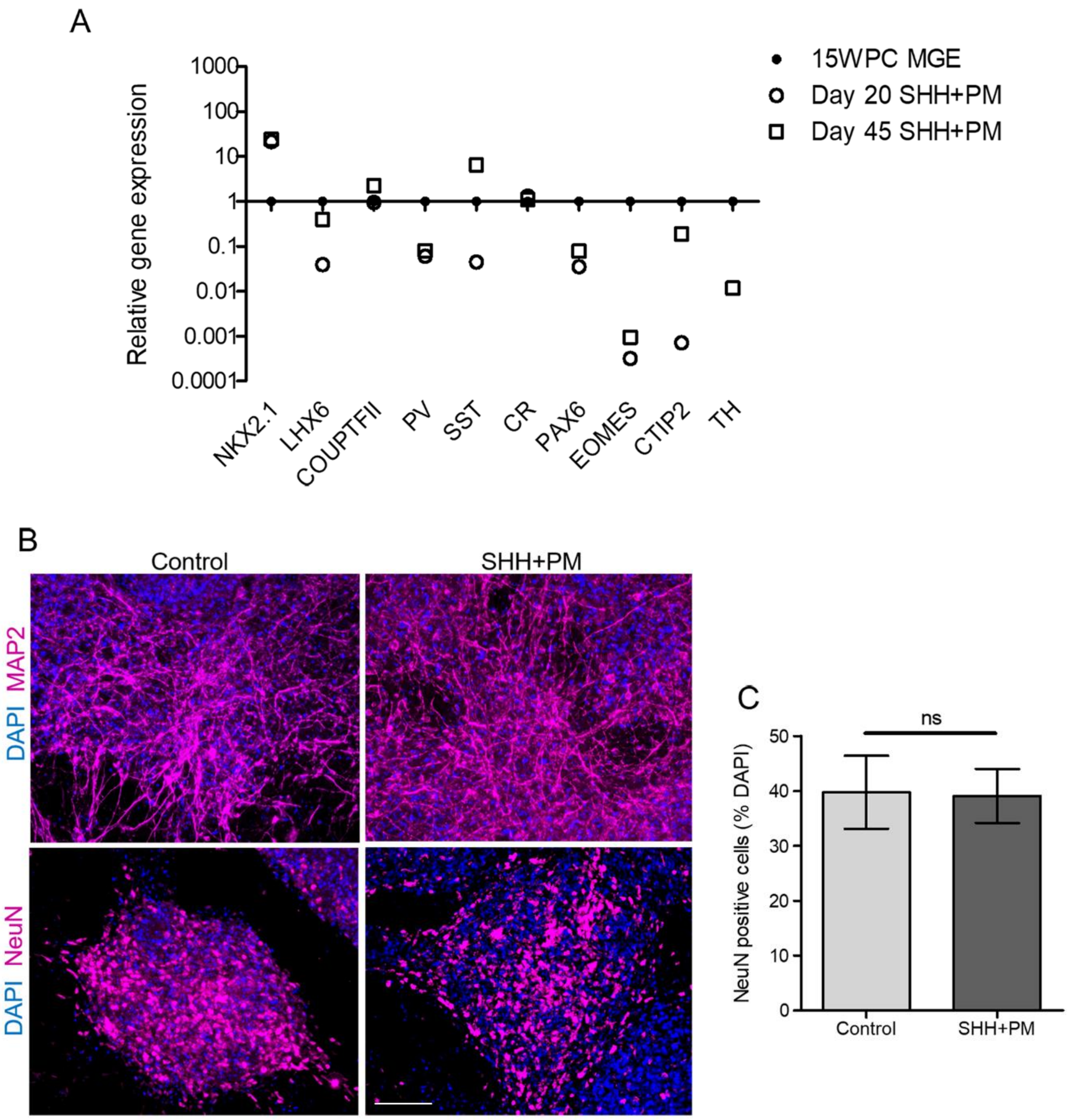


**Figure S1. Additional characterisation of hESC-neural derivatives**. (A) qPCR data in figure 1 presented as gene expression fold-change of SHH-treated relative to 15 weeks old human fetal MGE tissue. Data are presented as mean fold-change ± SEM from 3 independent replicates performed in a H7 subclone. Data on human fetal tissues was an average of two independent samples. (B) MAP2 and NeuN antibody staining of day 55 control and SHH+PM treated cultures, counterstained with DAPI (blue). A similar proportions of $MAP2^{+}$ and $NeuN^{+}$ cells were present in both conditions. (C) Counting data for NeuN staining. Graph shows mean +/- SEM. No significant difference between NeuN count between treatments ($p>0.05$ unpaired two-tailed t-test). Scale bar: 150μm.

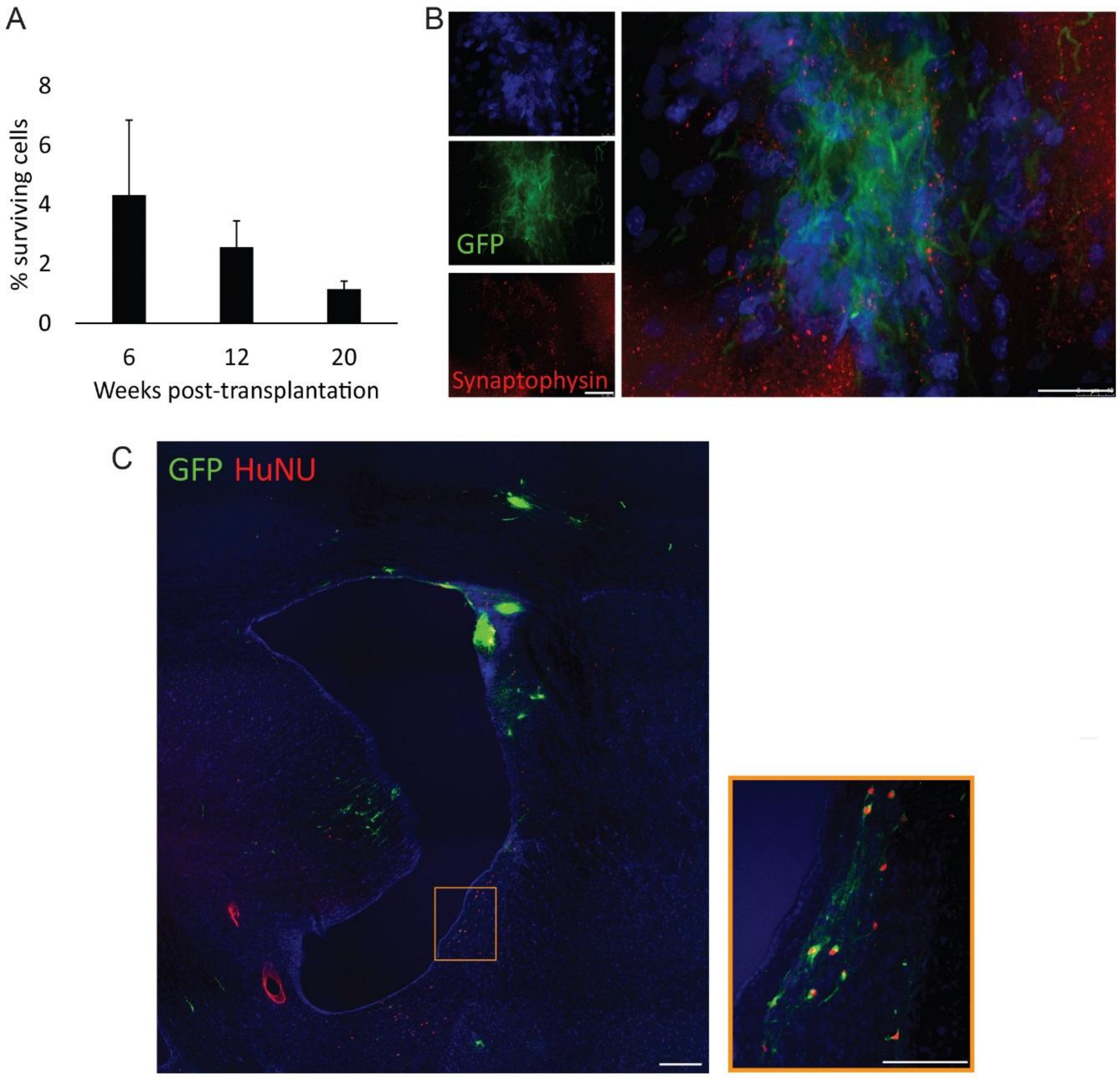


**Figure S2. Migration and synapse formation of grafted human neurons**

(A) Bar graph showing the mean ± SEM percentage of surviving cells (from $2 \times 10^5$ cells grafted) at each time point. (B) Immunocytochemistry image of synaptic marker Synaptophysin expressed within a clump of GFP+ grafted cells. (C) Tilescan immunocytochemistry image from 12 weeks post-transplantation showing potential migratory path of cells from striatum towards septum and hippocampus, with magnified yellow box to show HuNu/GFP co-expression. Scale bars: 100 $\mu$m.

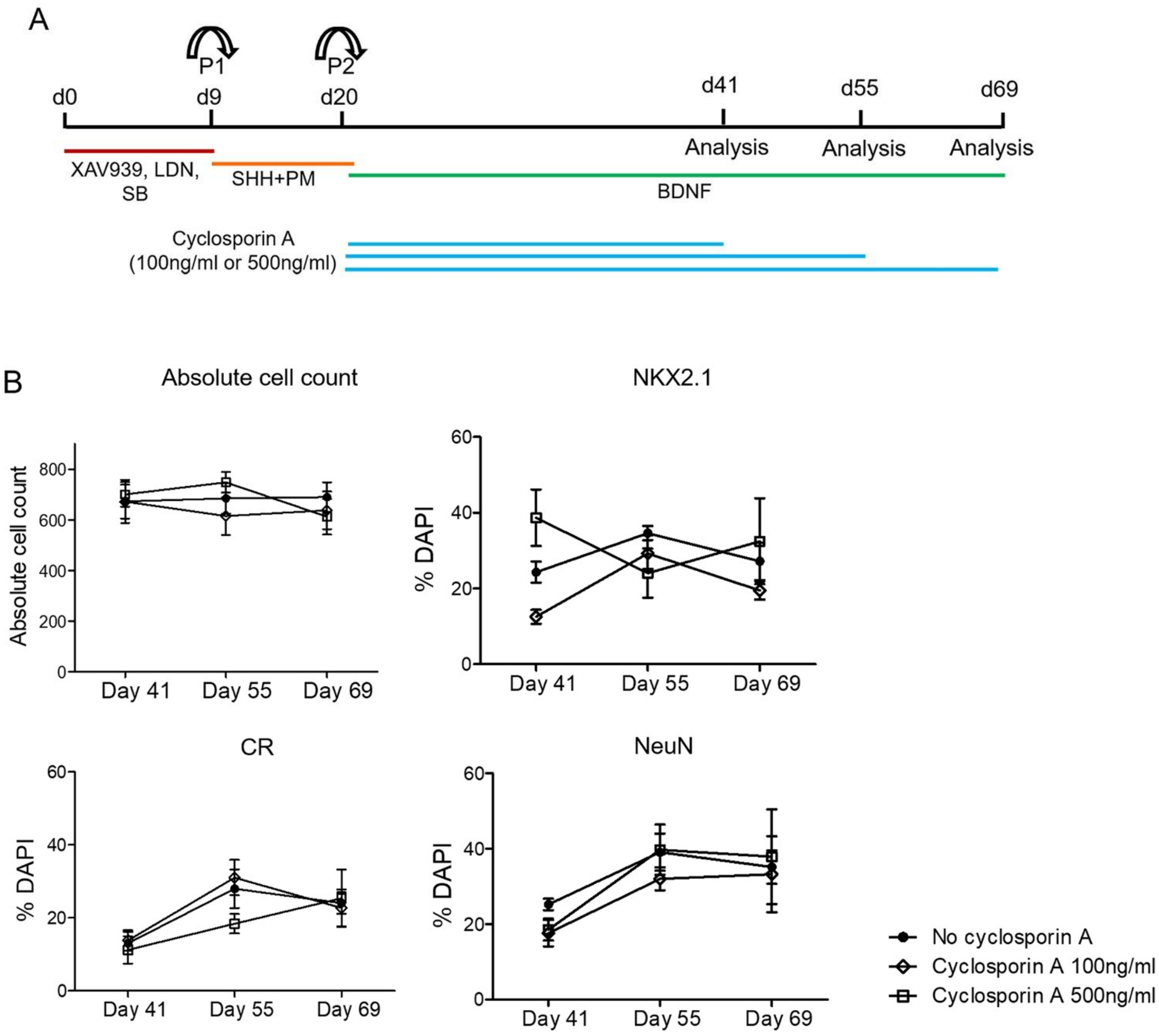


**Figure S3. Cyclosporin A treatment had no effect on cell proliferation/survival or the cell fate.** (A)Schematics of Cyclosporin A treatment. Cultures were exposed with or without cyclosporine A for 3 weeks (day 20 until day 41), 5 weeks (day 20 until day 55) and 7 weeks (day 20 until day 69) at either 100ng/ml or 500ng/ml cyclosporin A. Staining for each marker was carried out for all groups at indicated time point. No significant difference was found between cyclosporin A treated and control cultures (P>0.5, two-way ANOVA) for any of the markers. Data represent mean +/- SEM of three biological replicates for the markers indicated.

## Supplemental tables

### Table S1. qPCR data for Figures 1 and S1

| Time point | qPCR target gene | Mean fold-change | SEM | P value |
|---|---|---|---|---|
| D20 | *NKX2.1* | 3427.60 | 270.45 | 0.003 |
| | *LHX6* | 21.45 | 5.74 | 0.003 |
| | *COUP-TFII* | 0.83 | 0.08 | 0.142 |
| | *PAX6* | 0.01 | 0.00 | 0.001 |
| | *EMX1* | 0.01 | 0.01 | 0.022 |
| | *EOMES* | 0.01 | 0.00 | 0.001 |
| D45 | *NKX2.1* | 2729.88 | 0.62 | 0.003 |
| | *LHX6* | 241.65 | 0.15 | 0.001 |
| | *COUP-TFII* | 2.70 | 0.15 | 0.032 |
| | *PV* | 5.10 | 0.28 | 0.043 |
| | *SST* | 1.45 | 0.24 | 0.202 |
| | *CR* | 0.22 | 0.30 | 0.045 |
| | *PAX6* | 0.01 | 0.23 | 0.001 |
| | *EOMES* | 0.38 | 0.41 | 0.120 |
| | *CTIP2* | 0.20 | 0.28 | 0.033 |
| | *TH* | 0.57 | 0.35 | 0.832 |
| | *EphB1* | 2.38 | 0.19 | 0.051 |
| | *EphB3* | 2.26 | 0.22 | 0.070 |
| | *NRP1* | 2.99 | 0.20 | 0.034 |

**Table S2. Immunocytochemistry data for Figures 1 and S1**

| Time point | ICC target protein | Control | | SHH+PM | | P value |
|---|---|---|---|---|---|---|
| | | Mean | SEM | Mean | SEM | |
| D20 | NKX2.1 | 3.0 | 2.1 | 72.3 | 3.5 | 0.000 |
| | FOXG1 | 75.8 | 1.6 | 64.9 | 5.0 | 0.439 |
| | ASCL1 | 0.1 | 0.0 | 23.0 | 0.6 | 0.018 |
| | OLIG2 | 1.5 | 0.6 | 25.7 | 4.2 | 0.000 |
| | COUP-TFII | 22.2 | 3.9 | 22.0 | 4.7 | 0.169 |
| D60 | NKX2.1 | 7.2 | 2.2 | 42.1 | 5.1 | 0.000 |
| | SST | 1.3 | 0.5 | 6.3 | 1.3 | 0.000 |
| | PV | 0.0 | 0.0 | 1.5 | 0.9 | 0.116 |
| | CR | 3.3 | 0.8 | 3.9 | 0.4 | 0.620 |

**Table S3. Electrophysiology data for Figure 2**

| Measurement | Unit | D45 | | D60 | | P value |
|---|---|---|---|---|---|---|
| | | Mean | SEM | Mean | SEM | |
| Resting membrane potential | mV | -33.7 | 1.89 | -39.3 | 1.84 | 0.022 |
| Input resistance | MΩ | 1394.6 | 153.4 | 1368.1 | 168.1 | 0.545 |
| Membrane time constant | ms | 81.0 | 8.13 | 98.4 | 10.14 | 0.189 |
| Capacitance† | pF | 63.7 | 4.04 | 81.3 | 10.81 | 0.072 |
| Spontaneous event frequency | Hz | 0.43 | 0.08 | 0.45 | 0.15 | 0.451 |
| Evoked spike frequency | Hz | 9.39 | 2.05 | 7.05 | 2.21 | 0.778 |
| Spike amplitude | mV | 63.3 | 3.33 | 67.1 | 3.61 | 0.221 |
| Spike half-width | ms | 5.9 | 0.42 | 3.8 | 0.28 | 0.000 |
| Total neurite length | µm | 1786.1 | 166.1 | 2573.0 | 283.9 | 0.015 |
| Primary path length | µm | 207.7 | 46.4 | 220.4 | 41.8 | 0.420 |
| Branch length | µm | 1578.4 | 178.2 | 2352.6 | 301.6 | 0.022 |

†Equal variance not assumed

## Supplemental experimental procedures

### *HESC culture and differentiation*

HESCs – H7 and derivative lines (H7-tauGFP), H9 or iCas9-HUES9 (González *et al.* 2014) – were cultured on hESC-qualified Matrigel and kept in TeSR-E8 medium. Neural induction was initiated as previously described in (Arber et al., 2015) with additional ventral patterning based on (Maroof et al., 2010; Maroof et al., 2013). Briefly, cells were grown to 90% confluency in TeSR-E8 before switching to (as day 0) N2B27 medium supplemented with SB431542 (10 µM, Tocris), LDN193189 (100 nM, Sigma) and XAV939 (2 µM, Tocris) from day 0-9. SHH (200 ng/ml, C24II R&D) and Purmorphamine (1 µM, Millipore) were added from day 10 to day 18 and BDNF (10 ng/ml, Peprotech) from day 25 onwards. Cultures were passaged on days 9 and 20 using 0.02% EDTA and seeded onto plates coated with fibronectin (15 µg/ml) or poly-D-lysine (10 µg/ml) and laminin (10 µg/ml), respectively.

### *Derivation of H7-GFP cell line*

A mammalian expression vector CAG-tauGFP was used to generate a H7 derivative line constitutively expressing a tauGFP fusion protein(Pratt et al., 2000). The puromycin resistant gene (*pac*) is linked downstream of the *tauGFP* fusion gene via an internal ribosome entry site (IRES) to ensure that all puromycin resistan cells co-express tauGFP. Plasmid DNA was transfected into H7 hESCs using the Lonza 4D-Nucleofector as per manufacture instruction. 48 hours after nucleofection, cells were selected in the presence of 1µg/ml puromycin for 10 days. Surviving colonies were isolated and verified for stable GFP expression throughout neuronal differentiation. The best line (named H7-GFP) that exhibited high level and constitutive GFP expression in the derived neuronal cells was used for electrophysiology and transplantation experiments. The H7-GFP cells behaved similarly in interneuron differentiation compared to the H7 parental line and other two hESC lines tested.

### *Electrophysiology*

Primary mouse astrocytes were isolated from the cortices of postnatal day 7 C57BL/6 mouse pups and then seeded onto PDL-laminin-coated 13 mm glass coverslips. GFP-expressing neural progenitors were dissociated using Accutase (Thermo Fisher) on differentiation day 20 and seeded on top of the astrocytes at a density of 80,000 cells per $cm^2$. Cells were initially grown in a specialised culture

medium (BrainPhys) designed to aid functional maturation (Bardy et al., 2015) supplemented with a cocktail of growth factors and small molecules for 7 days followed by maintenance in BrainPhys medium alone (Telezhkin et al., 2016).

For whole cell patch clamp experiments, coverslips were transferred to a recording chamber maintained at room temperature (20-21°C) on the stage of an Olympus BX61W (Olympus) differential interference contrast (DIC) microscope and perfused at 2.5 ml/min with aCSF containing (in mM): 135 NaCl, 5 KCl, 1.2 MgCl2, 1.25 CaCl2, 10 D-glucose, 5 HEPES (Sigma), pH 7.4. GFP-expressing neurons were identified for recording by their fluorescence emission, which was viewed using a Rolera Bolt CMOS video rate camera (QImaging) following excitation by a 473 nm blue LED (CoolLED). Whole cell patch clamp recordings were performed using a MultiClamp 700B amplifier and pipettes with resistances of 4-8 MΩ when filled with an intracellular recording solution containing (in mM): 117 KCl (KGluc for voltage clamp), 10 NaCl, 11 HEPES, 2 Na2-ATP, 2 Na-GTP, 1.2 Na2-phosphocreatine, 2 MgCl2, 1 CaCl2 and 11 EGTA (Sigma), pH 7.2, supplemented with 0.1 AlexaFluor Hydrazide 555 (Thermo Fisher Scientific). Electrophysiological data were sampled at 20 kHz and filtered at 3 kHz using a Digidata 1550 analogue to digital converter and pClamp 10 software (Molecular Devices, USA). Series resistance was compensated using the bridge-balance and varied <20% during recordings. Recordings were not corrected for liquid junction potentials.

Resting membrane potential (RMP), input resistance ($R_N$), membrane time constant ($\tau$) and membrane capacitance ($C_m$) were measured in current clamp mode. RMP was measured using the mean baseline membrane potential over a 10 second sampling period immediately after establishing whole-cell access. $R_N$ was calculated using Ohm's law from the amplitude of the steady-state voltage deflection in response to a 1 second hyperpolarizing current injection (-10 pA). $C_m$ was estimated according to $Cm = \tau_m/R_N$ where $\tau_m$ was calculated by fitting a single exponential function to the mean voltage response evoked by a series of hyperpolarizing current pulses (-10 pA, 1 s). For evoked activity measurement, cells were injected with current steps of 20 pA between -100 and +200 pA (1 s) to measure voltage responses. The properties of single action potentials were calculated from the first spike evoked by a rheobase current injection step.

Neurotransmitter-evoked currents were measured using voltage clamp recordings. Brief pulses (30 ms) of glutamate or GABA (100 µM) were applied using a custom built pressure application system via a second pipette positioned close to the soma of the recorded neuron. The mean neurotransmitter-evoked current was calculated as the average of 5 pressure applications delivered at 30 s intervals.

For morphological analysis, neurons were filled with Alexa 555 during whole cell recordings and their soma and complete dendritic tree imaged using a Rolera Bolt CMOS video rate camera (QImaging). Acquired fluorescence images were manually traced post-hoc using Neurolucida 360 (MBF biosciences, USA) and analysed using Neurolucida Explorer.

Data were analysed using Clampfit 10 software (Molecular Devices) and then exported to and plotted using Origin 9 (OriginLab).

### *Quantitative real-time PCR (qPCR)*

Total RNA was extracted using Tri reagent treated with TURBO DNase. cDNA was generated using qScript cDNA synthesis kit. qPCR was performed with Mesa Green qPCR master mix with specific primers listed in table below and dissociation curves were recorded to check for amplification specificity. Cq values were normalised to two housekeeping reference genes and changes in expression calculated using the 2-∆∆CT method. Data shown are mean±SEM of three biological replicates performed in H7 cells and each sample was measured in triplicate on a CFX Connect Real Time PCR machine.

### *Immunocytochemistry*

Cultured cells were washed with PBS and fixed in 3.7% PFA for 15 minutes at room temperature. For nuclear stains, fixed cells underwent 5 minute methanol washes of ascending then descending methanol dilutions in PBS (33% and 66% at room temperature, then 100% at -20°C). Cells were blocked for 30 minutes in PBS-T (0.3% Triton-X-100) with 2% BSA and 5% donkey serum. Primary antibodies were added in PBS-T with 3% donkey serum and left overnight at 4°C. Secondary antibodies (AlexaFluor anti-donkey 488, 555, 647; Life Technologies) were added in PBS-T and left for 1 hour at room temperature. DAPI (Molecular Probes) was used to counterstain cell nuclei. Staining was preserved using DAKO fluorescent mounting medium (Life Technologies). Samples were imaged on a Leica DMI6000b

fluorescent microscope and cells were counted manually with ImageJ (cytoplasmic markers) or using automated Cell Profiler (nuclear markers). Cell counts from at least 3 independent experiments were used for statistical analysis.

### *Immunohistochemistry*

Coronal sections were cut on a freezing microtome to 40 µm thickness in a 1:12 series. Floating sections were blocked in Triton-X-100-TBS with 3% donkey serum for 1 hour, then incubated in TXTBS with 1% donkey serum and primary antibodies overnight. Sections were washed 3 times in TBS then incubated in TBS with secondary antibodies (1/200, AlexaFluor 488, 555, 594 and 647) overnight. After 3 more TBS washes sections were mounted onto glass slides, air dried, cover-slipped with VectaShield hardset antifade mounting medium with DAPI (Vector Labs) and stored at 4°C. Slides were imaged using a Leica DM6000B microscope. Neurolucida 360 software (MBF Bioscience) was used to manually reconstruct imaged neurons and conduct Sholl analysis and path length analysis.

### *Transplantation*

All animal work was done in compliance with the European Directive 2010/63/EU on the protection of animals used for scientific purposes. All surgical procedures were carried out under isoflurane anaesthesia using a neonatal adaptor on a stereotaxic frame. Postnatal day 2 Sprague Dawley pups (n=15) were injected with $2 \times 10^5$ cells in a volume of 1 µl using a Hamilton syringe. Injection coordinates targeted the right striatum (0.9 mm anterior and 1.8 mm lateral to bregma, 2.0 mm below the dura; 0.7 mm anterior and 1.9 mm lateral to bregma, 2.9 mm below the dura). After immune rejection was observed in a pilot study, animals were given daily intraperitoneal Cyclosporine A injections (10 mg/kg; Sandimmun) from weaning onwards, and were sacrificed at 6, 12 and 20 weeks (n=6, 5 and 4) post-surgery by transcardial perfusion with 4% PFA and post-fixed for a further 4 hours.

*List of qPCR primers*

| Gene | Forward primer | Reverse primer |
|---|---|---|
| GAPDH | ATGACATCAAGAAGGTGGTG | CATACCAGGAAATGAGCTTG |
| β-ACTIN | TCACCACCACGGCCGAGCG | TCTCCTTCTGCATCCTGTCG |
| NKX2.1 | CGCATCCAATCTCAAGGAAT | TGTGCCCAGAGTGAAGTTTG |
| LHX6 | GACGACATCCACTACACCCC | GGCCCATCCATATCGGCTTT |
| COUPTFII | GGAGAAGCTCAAGGCACTGCA | CCTGCAAGCTTTCCACATGGG |
| PAX6 | AATAACCTGCCTATGCAACCC | AACTTGAACTGGAACTGACACAC |
| EMX1 | ACCGGAGGACAAAGTACAAAC | TAGTCATTGGAGGTGACATCG |
| EOMES | CTGCCTACCAAAACACCGATATTAC | AGCGGGCTTGAGGTAAAGTG |
| PVALB | AAAGAGTGCGGATGATGTGAAG | ACCCCAATTTTGCCGTCCC |
| SST | GCTGCTGTCTGAACCCAAC | CGTTCTCGGGGTGCCATAG |
| CR | TCAGAGATGTCCCGACTCCTG | GCCGCTTCTATCCTTGTCGTAA |
| CTIP2 | CTCCGAGCTCAGGAAAGTGTC | TCATCTTTACCTGCAATGTTCTCC |
| TH | GAGTACACCGCCGAGGAGATTG | GCGGATATACTGGGTGCACTGG |
| EPHB1 | GCACATCTCTGGTGATTGCTC | ACGCTGTTCTCAGGCTCATAG |
| EPHB3 | GGCCATAGCCTATCGGAAGT | TCCCAGTAGGGTCGCTCTC |
| NRP1 | AAGGTTTCTCAGCAAACTACAGTG | GGGAAGAAGCTGTGATCTGGTC |

### *List of primary antibodies*

| Antigen | Species | Supplier | Catalogue number | Dilution | |
|---|---|---|---|---|---|
| | | | | Cells | Rat tissue |
| Calretinin | rabbit | Swant | 7697 | 1/500 | 1/1000 |
| ChAT | goat | Millipore | AB144 | 1/100 | 1/200 |
| FOXG1 | rabbit | Abcam | 18259 | 1/250 | |
| GAD67 | mouse | Millipore | mab5406 | 1/500 | 1/500 |
| GFP | rabbit | Invitrogen | | 1/500 | 1/1000 |
| HuNu | mouse | Millipore | mab1281 | 1/250 | 1/1000 |
| ASCL1 | mouse | BD | 556604 | 1/500 | |
| Nestin | mouse | Neuromics | | 1/300 | 1/300 |
| NeuN | mouse | Millipore | mab377 | 1/250 | 1/500 |
| NKX2.1 (TTF1) | rabbit | Abcam | ab40880 | 1/1000 | 1/1000 |
| NPY | rabbit | Immunostar | 22940 | 1/200 | 1/250 |
| Oct4 | goat | Santa Cruz | sc8628 | 1/500 | |
| OLIG2 | goat | R&D | | 1/200 | |
| Parvalbumin | mouse | Sigma | p3088 | 1/100 | 1/100 |
| Somatostatin | rabbit | Millipore | | 1/50 | |
| Somatostatin | rat | Millipore | | 1/50 | 1/100 |

## References

Arber, C., Precious, S.V., Cambray, S., Risner-Janiczek, J.R., Kelly, C., Noakes, Z., Fjodorova, M., Heuer, A., Ungless, M.A., Rodriguez, T.A., *et al.* (2015). Activin A directs striatal projection neuron differentiation of human pluripotent stem cells. Development *142*, 1375-1386.

Bardy, C., van den Hurk, M., Eames, T., Marchand, C., Hernandez, R.V., Kellogg, M., Gorris, M., Galet, B., Palomares, V., Brown, J., *et al.* (2015). Neuronal medium that supports basic synaptic functions and activity of human neurons in vitro. Proceedings of the National Academy of Sciences of the United States of America *112*, E2725-2734.

Maroof, A.M., Brown, K., Shi, S.-H., Studer, L., and Anderson, S.A. (2010). Prospective Isolation of Cortical Interneuron Precursors from Mouse Embryonic Stem Cells. Journal of Neuroscience *30*, 4667-4675.

Maroof, A.M., Keros, S., Tyson, J.A., Ying, S.W., Ganat, Y.M., Merkle, F.T., Liu, B., Goulburn, A., Stanley, E.G., Elefanty, A.G., *et al.* (2013). Directed differentiation and functional maturation of cortical interneurons from human embryonic stem cells. Cell stem cell *12*, 559-572.

Pratt, T., Sharp, L., Nichols, J., Price, D.J., and Mason, J.O. (2000). Embryonic stem cells and transgenic mice ubiquitously expressing a tau- tagged green fluorescent protein. Developmental biology *228*, 19-28.
Telezhkin, V., Schnell, C., Yarova, P., Yung, S., Cope, E., Hughes, A., Thompson, B.A., Sanders, P., Geater, C., Hancock, J.M.*, et al.* (2016). Forced cell cycle exit and modulation of GABAA, CREB, and GSK3beta signaling promote functional maturation of induced pluripotent stem cell-derived neurons. American journal of physiology. Cell physiology *310*, C520-541.
