# Supplementary material for: Human Pluripotent Stem Cell-Derived Striatal Interneurons: Differentiation and Maturation In Vitro and in the Rat Brain
Source: Stem Cell Reports. 2019 Jan 17;12(2):191–200. doi: 10.1016/j.stemcr.2018.12.014 (PMC6373547; doi:10.1016/j.stemcr.2018.12.014)
Supplement: Document S2. Article plus Supplemental Information [file mmc2.pdf]

Stem Cell Reports
Report
ISSCR
OPEN ACCESS

Human Pluripotent Stem Cell-Derived Striatal Interneurons: Differentiation and Maturation *In Vitro* and in the Rat Brain

Zoe Noakes,[1,2,*] Francesca Keefe,[1,2] Claudia Tamburini,[1] Claire M. Kelly,[2] Maria Cruz Santos,[1] Stephen B. Dunnett,[2] Adam C. Errington,[1] and Meng Li[1,2,*]
[1]Neuroscience and Mental Health Research Institute, School of Medicine, Cardiff University, Cardiff CF24 4HQ, UK
[2]School of Biosciences, Cardiff University, Cardiff CF10 3AX, UK
*Correspondence: noakesz@cardiff.ac.uk (Z.N.), lim26@cardiff.ac.uk (M.L.)
https://doi.org/10.1016/j.stemcr.2018.12.014

SUMMARY

Striatal interneurons are born in the medial and caudal ganglionic eminences (MGE and CGE) and play an important role in human striatal function and dysfunction in Huntington's disease and dystonia. MGE/CGE-like neural progenitors have been generated from human pluripotent stem cells (hPSCs) for studying cortical interneuron development and cell therapy for epilepsy and other neurodevelopmental disorders. Here, we report the capacity of hPSC-derived MGE/CGE-like progenitors to differentiate into functional striatal interneurons. *In vitro*, these hPSC neuronal derivatives expressed cortical and striatal interneuron markers at the mRNA and protein level and displayed maturing electrophysiological properties. Following transplantation into neonatal rat striatum, progenitors differentiated into striatal interneuron subtypes and were consistently found in the nearby septum and hippocampus. These findings highlight the potential for hPSC-derived striatal interneurons as an invaluable tool in modeling striatal development and function *in vitro* or as a source of cells for regenerative medicine.

INTRODUCTION

The medial ganglionic eminence (MGE) and caudal ganglionic eminence (CGE) give rise to cortical, striatal and hippocampal interneurons, as well as globus pallidus projection neurons and cholinergic basal forebrain neurons. These cells are crucial for cortical and basal ganglia function; and their dysfunction has been implicated in diseases such as epilepsy, schizophrenia, autism, Huntington's disease (HD), and dystonia (Lewis, 2012; Powell et al., 2003; Reiner et al., 2013; Zikopoulos and Barbas, 2013). Given the correct patterning cues, human pluripotent stem cells (hPSCs) can differentiate into any cell type in the body, providing an excellent *in vitro* tool for the study of human neural development and function. On this topic, there has been much interest in using hPSCs to generate cortical or hippocampal GABAergic interneurons (Cambray et al., 2012; Kim et al., 2014; Maroof et al., 2010; Nicholas et al., 2013), and some efforts to produce cholinergic forebrain neurons (Bissonnette et al., 2011; Crompton et al., 2013). However, little attention has been paid to striatal interneurons despite their important role in dystonia and HD (Capetian et al., 2014; Reiner et al., 2013).

While interneurons comprise only 5%–10% of rodent striatal neurons, they make up more than 20% of primate striatal neurons, suggesting a more important role in primates than in rodents (Graveland and DiFiglia, 1985; Wu and Parent, 2000). The remaining striatal population are the projecting medium spiny neurons (MSNs), born in the adjacent lateral ganglionic eminence (LGE). Proof-of-principle studies showing functional improvement in HD animal models have used whole ganglionic eminence (WGE) comprising both LGE and MGE fetal tissue (Kendall et al., 1998; Palfi et al., 1998). Interneurons will likely be essential for modeling striatal function with hPSCs, and may help transplanted hPSC-derived LGE-like cells to differentiate into MSNs and integrate *in vivo* for the treatment of HD.

Striatal interneurons fall into four main subtypes with distinct molecular and functional characteristics. Parvalbumin (PV)- and somatostatin (SST)-expressing GABAergic interneurons and choline acetyltransferase (ChAT)-expressing cholinergic interneurons are born in the MGE marked by transcription factor NKX2.1. Most calretinin (CR) interneurons arise from COUP-TFII-expressing progenitors in the CGE (Butt et al., 2005; Marin et al., 2000). The only known molecular profile that reliably distinguishes MGE-derived striatal and cortical interneurons *in vivo* is co-expression of NKX2.1 and LHX6. While cortical interneurons switch off expression of NKX2.1 on post-mitotic upregulation of LHX6, striatal interneurons maintain expression of both transcription factors into adulthood (Nobrega-Pereira et al., 2008).

The common developmental origin of cortical and striatal interneurons indicates that differentiating hPSCs toward cortical interneurons should also produce striatal interneurons. Here we show the production of GABAergic interneurons of each subtype *in vitro* from human embryonic stem cells (hESCs), which demonstrated maturing electrophysiological properties. Upon transplantation into the neonatal rat striatum, hESC-derived neural progenitors differentiated into striatal CR and cholinergic

This is an open access article under the CC BY license (http://creativecommons.org/licenses/by/4.0/).

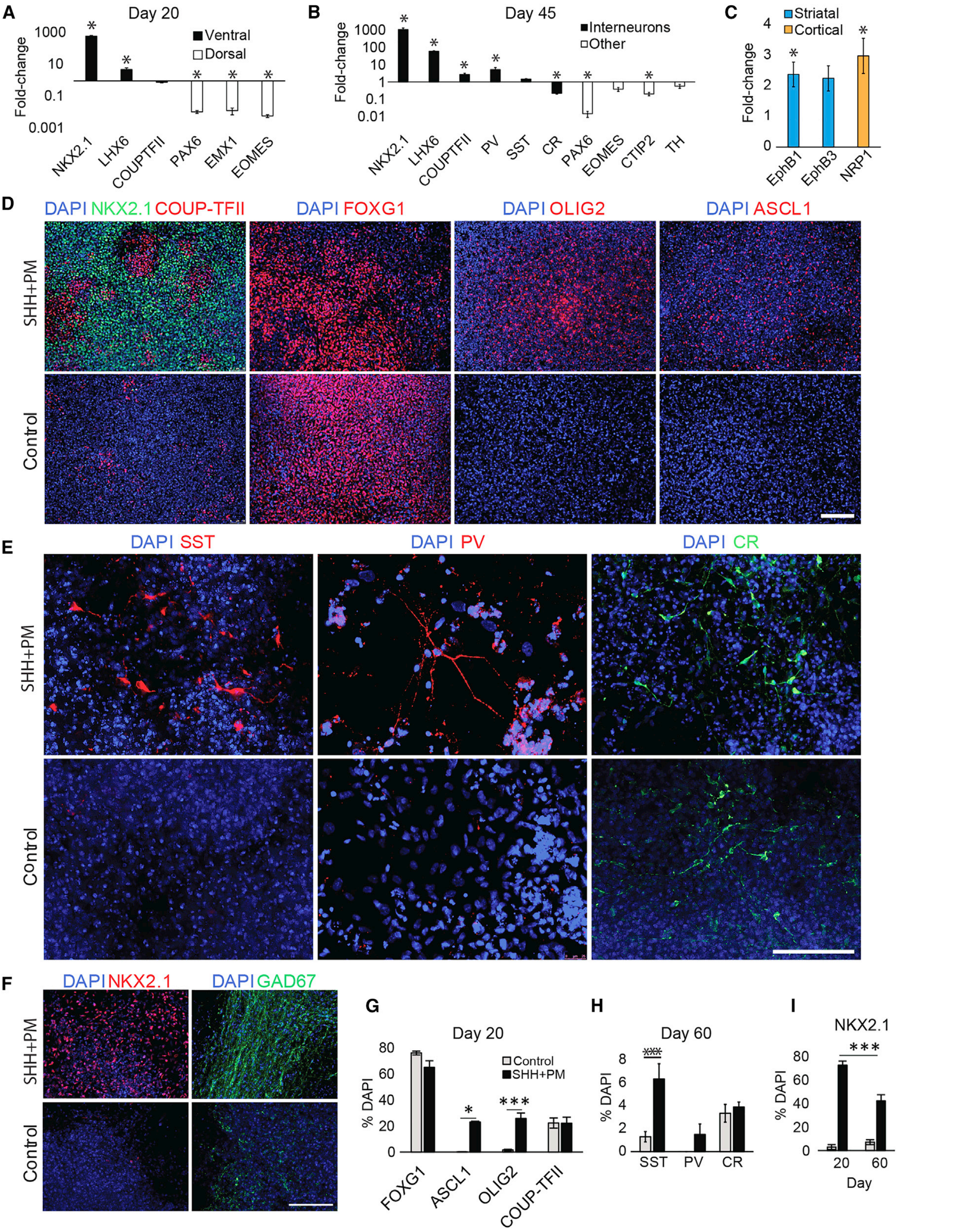

(legend on next page)

interneurons and showed region-specific morphology depending on where they settled.

RESULTS

HESC-Derived MGE- and CGE-like Progenitors Give Rise to Striatal Interneuron-like Cells *In Vitro*

hESCs seeded in a monolayer underwent neural induction by dual-Smad inhibition, with rostral fate facilitated by WNT signaling inhibitor, XAV939. This was followed by combinatorial treatment of sonic hedgehog (SHH) and purmorphamine to induce ventral forebrain identity. We performed qPCR analyses at days 20 (D20) and 45 (D45) of differentiation. At D20, SHH-treated cultures showed a marked increase of MGE markers *NKX2.1* and *LHX6*, and decrease of dorsal forebrain markers *PAX6*, *EMX1*, and *EOMES* compared with untreated controls (Figure 1A; Table S1). Little change was observed for CGE marker *COUP-TFII* between conditions. At D45, *NKX2.1*, and *LHX6* expression remained highly elevated in SHH-treated cultures, and COUP-TFII expression significantly increased to nearly three times that of control cultures (Figure 1B). Expression of interneuron subtype marker *PV* was five times higher in SHH-treated samples, but the marginal increase in *SST* expression was not significant. In contrast, *CR* expression was significantly lower in SHH-treated cultures, falling to 20% of that of controls. Expression of genes specific to regions other than the MGE or CGE were reduced or similar in SHH-treated cultures compared with controls. The transcript levels of MGE and post-mitotic interneuron marker genes in SHH-treated cultures were higher at D45 than those of D20; and were either similar or higher than those of 15-gestational-week human fetal MGE, apart from *LHX6* and *PV* (Figure S1A).

Striatal and cortical interneurons express distinct guidance molecules such that they respond differently to migratory cues and settle in the striatum or cortex (Nobrega-Pereira et al., 2008; Villar-Cervino et al., 2015). *EPHB1* and *EPHB3*—expressed by striatal interneurons—and cortical interneuron marker *NRP1*, showed increased mRNA levels in SHH-treated cultures compared with controls (Figure 1C; Table S1). This shows that SHH treatment led to increased transcription of MGE- and CGE-derived interneuron genes, and notably elevated levels of mRNA specific to both striatal and cortical interneuron guidance molecules.

Preferential induction of MGE fate in SHH-treated cultures was confirmed at the protein level by immunostaining of interneuron and progenitor markers. Most cells in D20 SHH-treated cultures expressed NKX2.1, and around a quarter of cells expressed ASCL1 and OLIG2 (Figures 1D, 1G, and 1I; Table S2). COUP-TFII expression was around 22% in both conditions, but in SHH-treated cultures its expression pattern appeared opposite to that of NKX2.1, with few cells expressing both proteins (Figure 1D). No statistical difference was found in the number of cells expressing FOXG1, a marker for all forebrain neural progenitors (Figures 1D and 1G). At D60, there was a higher proportion of SST$^+$ cells in those treated with SHH (SHH 6.3% ± 1.3%, Ctrl 1.3% ± 0.5%, $p < 0.001$) (Figures 1E and 1H). PV$^+$ cells were detected in three out of six experiments in SHH-treated cultures (1.5% ± 0.9%) but never observed in control cultures. CR was expressed in 3%–4% of cells in both conditions. SHH-treated cultures maintained a greater proportion of NKX2.1$^+$ cells at D60, but presented a significant drop of 30% compared with D20 (Figures 1F and 1I). GAD67$^+$ cells were widely observed in SHH-treated cultures, in visibly greater numbers than in controls (Figure 1F). Comparable numbers of MAP2$^+$ and NeuN$^+$ neurons were detected in the control and SHH-treated cultures (Figure S1B), suggesting that SHH treatment did not affect overall neuronal production. Together, these results confirm the generation of neurons resembling cortical and striatal GABAergic interneurons of different subtypes.

HESC-Derived Interneurons Develop Mature Electrophysiological and Morphological Properties *In Vitro*

We next assessed the functional maturation of hESC-derived neurons using whole-cell patch-clamp electrophysiology (Figure 2A). HESC-neural derivatives were

Figure 1. Differentiation of hESCs into Striatal and Cortical Interneurons *In Vitro*

(A–C) qPCR data presented as gene expression fold-change of SHH-treated samples relative to untreated controls on days 20 (A) and 45 (B) on a logarithmic y axis. Day 45 samples were also analyzed for their expression of cortical and striatal interneuron-specific markers (C). Data are presented as mean fold-change ± SEM from three independent experiments performed in H7 cells. *$p < 0.05$, two-sample t test with equal variance not assumed.

(D–F) Representative immunocytochemistry images of H7 control and SHH-treated cultures at days 20 (D) and 60 (E and F). Scale bars, 100 μm.

(G–I) Images were counted for MGE and CGE progenitor markers (G and I) and interneuron subtype markers (H and I). Data presented are mean ± SEM from independent experiments performed in H7 (n = 3), H7-tauGFP (n = 2), H9 (n = 1), and iCas9 (n = 1) for day 20 and H7 (n = 3) and H7-tauGFP (n = 2) for day 60.

*$p < 0.05$, ***$p < 0.001$, one-way ANOVA (COUP-TFII, SST, CR), Kruskal-Wallis test (FOXG1, ASCL1, OLIG2, PV), and two-way ANOVA with *post hoc* Bonferroni (NKX2.1).

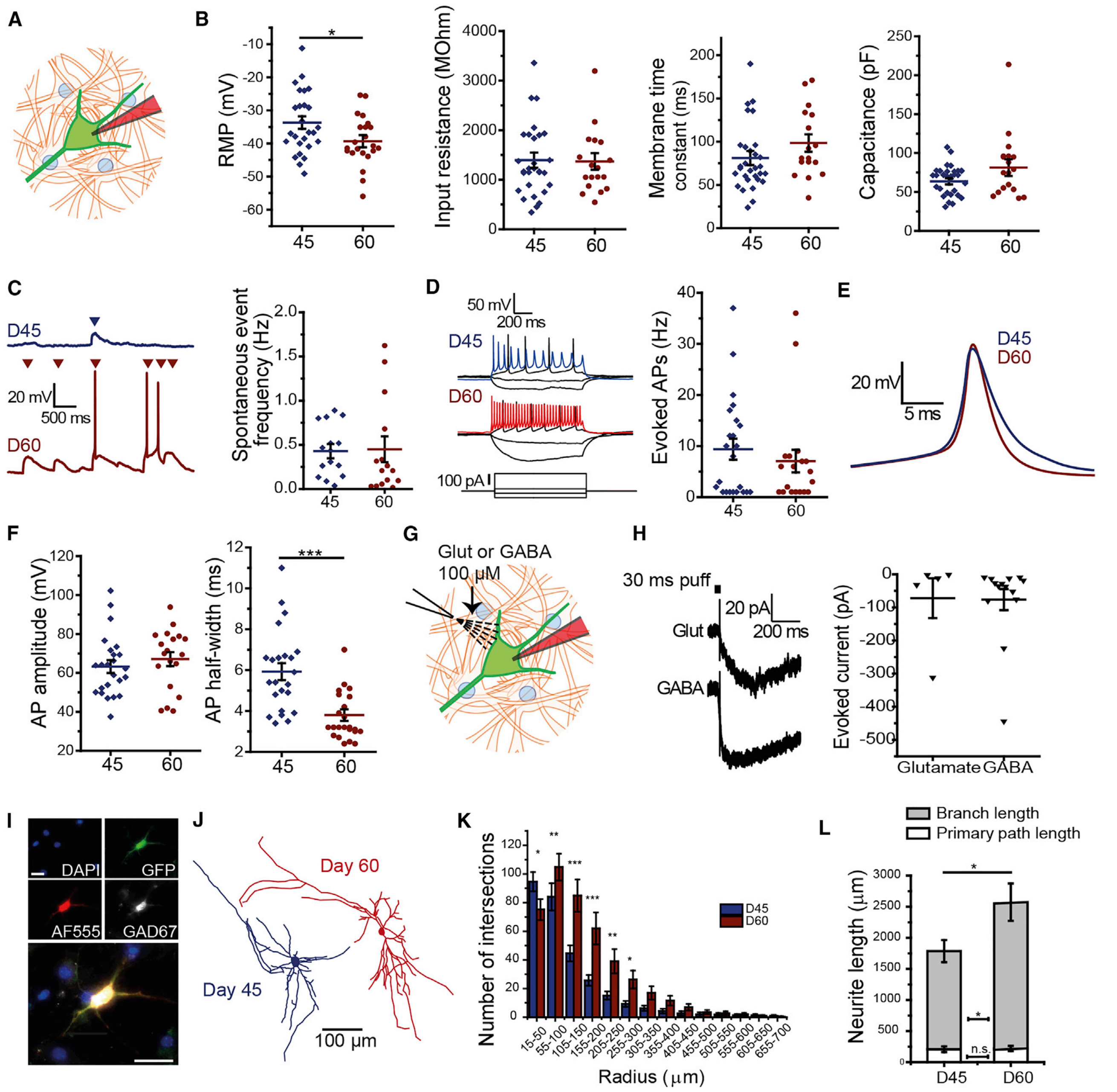

Figure 2. hESC-Derived MGE/CGE-like Progenitors Become Functional GABAergic Neurons

(A) Schematic of whole-cell patch clamp of H7-tauGFP^{+} neurons co-cultured with primary mouse astrocytes.

(B) Basic membrane properties at days 45 (n = 25) and 60 (n = 16–19). RMP, resting membrane potential.

(C) Representative traces of spontaneous activity (left). Post-synaptic potentials and action potentials were counted over 2 min (right: D45, n = 14; D60, n = 14).

(D) Representative traces of evoked activity from current injection steps (left). The maximum number of evoked spikes was quantified in each cell (right: D45, n = 23; D60, n = 19).

(E) Overlaid averaged traces of all D45 (blue, n = 15) and D60 (red, n = 16) first evoked spikes.

(F) Amplitude and half-width of first evoked spikes.

(G) Schematic illustrating second pipette for focal application of glutamate or GABA onto patched cell.

(H) Representative traces showing glutamate- (Glut, 100 μM) and GABA (100 μM)-evoked currents (left) and their quantification (right: Glut, n = 5; GABA, n = 14).

(legend continued on next page)

co-cultured with mouse astrocytes from passage 2 to promote neuronal maturation. An H7 derivative line that constitutively expresses a cytoplasmic TauGFP fusion protein was used for easier identification of human neurons (Pratt et al., 2000). First, using current clamp we measured intrinsic membrane properties at differentiation D45 and D60. The mean resting membrane potential decreased significantly, indicating functional maturation of the cells (Figure 2B; Table S3). However, there was no significant change in input resistance, membrane time constant, or capacitance of the cells.

To assess the intrinsic excitability of our hESC-derived neurons and their ability to form functional synapses, we calculated the frequency of spontaneous events (action potentials and post-synaptic potentials) and evoked spikes (Figures 2C and 2D; Table S3). Most cells displayed spontaneous activity and fired evoked trains of action potentials, but there was no significant change in the mean frequency of either over time. However, comparison of individual action potential kinetics revealed a 35% reduction in mean spike half-width—indicating an increase in the number of voltage-gated ion channels in the cell membrane—despite only a modest increase in mean spike amplitude (Figure 2F).

Striatal neurons are subject to both glutamatergic input from the cortex and thalamus, and GABAergic input from local connections. We tested the effects of focally applied glutamate and GABA in our D60 neurons using voltage clamp (Figure 2G). Held at −45 mV, glutamate evoked inward currents in three out of five cells (Figure 2H; Table S3). At a holding potential of −70 mV, all cells responded to GABA with an inward current driven primarily by $GABA_A$ receptors, verified by blocking with picrotoxin.

Finally, we investigated the molecular and morphological development of the hESC-derived neurons. *Post hoc* immunostaining confirmed that 60.6% of GFP^+ cells in the cultures expressed GAD67 (Figure 2I). During patch-clamp recordings, neurons were filled with Alexa Fluor 555 (100 μM) and imaged *in situ* for reconstruction using Neurolucida 360 (Figure 2J). Sholl analysis revealed significantly increased neurite complexity at D60, shown by a greater number of intersections up to 300 μm (Figure 2K). The significant difference in total neurite length was driven entirely by the growth of branches, while mean primary neurite length remained constant (Figure 2L; Table S3). Together, these data show that hESCs can differentiate into functional, morphologically complex neurons expressing relevant neurotransmitter receptors *in vitro*, making them a suitable platform with which to study neural functional development.

Transplanted hESC-Derived MGE/CGE-like Progenitors Give Rise to Striatal Interneuron-like Cells in Rat Striatum

We next explored the potential of the cells to adopt a striatal interneuron phenotype *in vivo*. D20 MGE/CGE-like progenitors, derived from the GFP^+ H7 line, were transplanted into the right striatum of rat pups immune-suppressed with cyclosporine A. At 6 weeks post-transplantation (WPT), GFP^+ cells were found in the striatum, septum, and hippocampus of four out of six recipients, and were confirmed to always co-label for HuNu despite some variation in GFP brightness (Figures 3A and S2). Neuronal morphology complexified over time, with a significant increase in the number of primary neurites by 20 WPT in the striatum and septum (Figures 3B and 3C). Cells in the striatum and septum also developed a significantly greater number of branch points and neurite terminations, indicating a difference in morphological development between cells that settled in the different regions (Figure 3C). These observations suggest that environmental cues had an impact on neuronal morphology, and that transplanted cells integrated themselves structurally within the host brain.

Immunostaining of the grafted brains revealed that around half of surviving cells had differentiated into post-mitotic neurons by 6 WPT, demonstrated by NeuN staining, which did not change at 12 or 20 WPT (Figures 4 and S2). Consistent with this, we observed Nestin staining in more than 40% of GFP^+ cells at 20 WPT, suggesting protracted neuronal differentiation *in vivo* (Figure 4C). NKX2.1 expression remained stable in a third of GFP^+ cells over time, and there was no difference across the brain regions in which cells settled (Figure 4). Again this indicates that many cells had differentiated by 6 WPT, and either that a pool of cells remained $NKX2.1^+$ progenitors, or that they maintained its expression—as would mature striatal interneurons in normal development (Nobrega-Pereira et al., 2008) We observed $synaptophysin^+$ puncta confirming the presence of synapses on transplanted cells,

(I) *Post hoc* immunocytochemistry of AF555-filled neurons to confirm GAD67 (white) expression. Scale bars, 15 μm.
(J) AF555-filled neurons were imaged and traced *post hoc* in Neurolucida.
(K) Quantification of Sholl analysis intersections compared by two-way ANOVA with *post hoc* Bonferroni correction.
(L) Total neurite length was divided into primary path length (white) and branch length (gray) (D45, n = 15; D60, n = 18). n represents number of cells from three independent experiments and all data plots show mean ± SEM of the cells recorded. All statistical analyses except for (K) were done by two-sample t test.
$^*p < 0.05$, $^{**}p < 0.01$, $^{***}p < 0.001$.

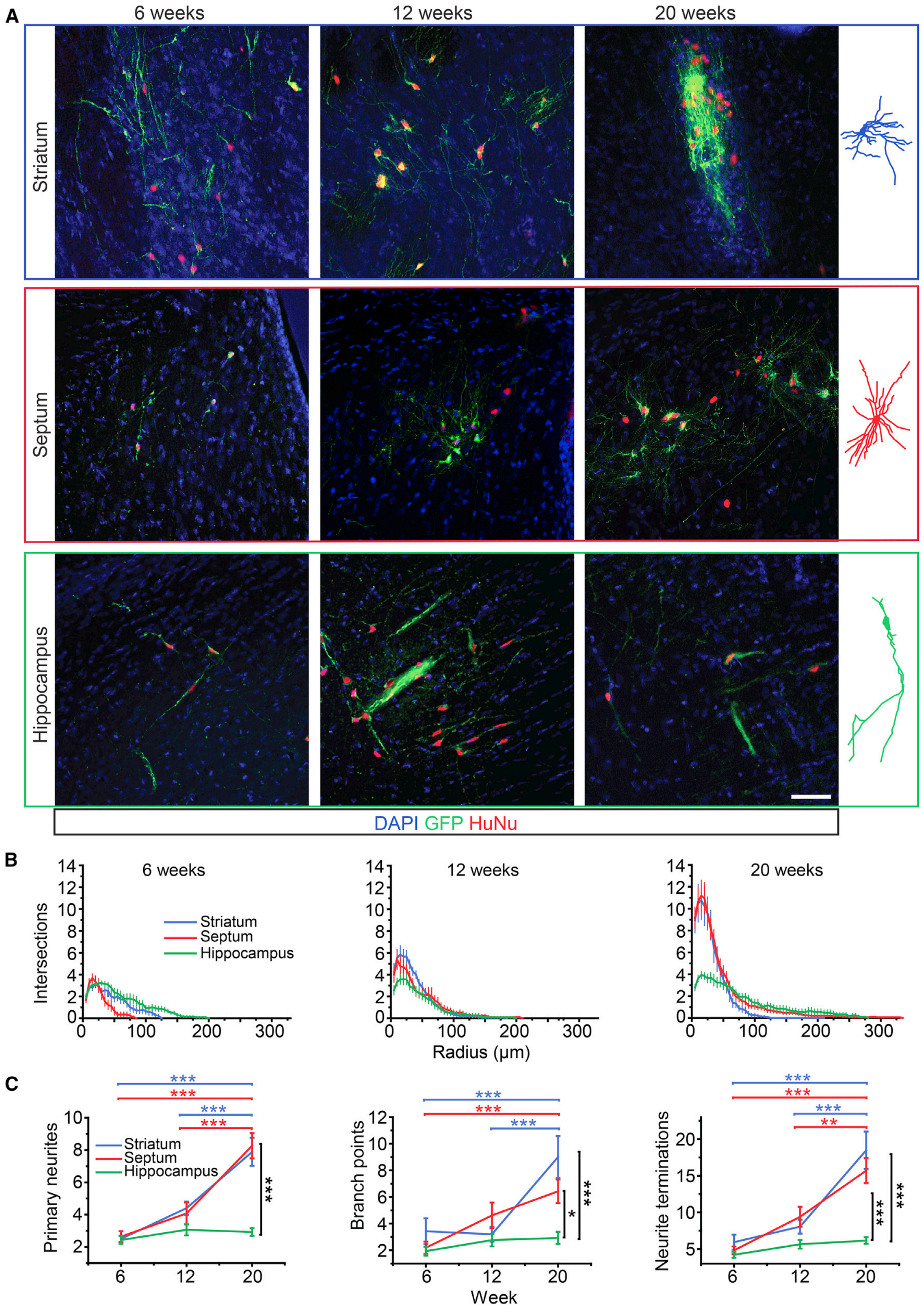

(legend on next page)

although we cannot conclude whether these were from host innervation (Figure S2).

Looking at interneuron subtype-specific markers, CR was widely expressed throughout the grafts at 12 and 20 WPT, both in the striatal graft cores and in morphologically mature neurons in the striatum, septum, and hippocampus (Figures 4B and 4C). Neither PV nor SST expression were observed in any GFP+ cells, unlike sister cultures that matured *in vitro* (Figure 1). However, ChAT—a cholinergic interneuron marker not observed *in vitro* but normally present in the striatum—was expressed in a small number of striatal GFP+ cells at 12 and 20 WPT (Figures 4B and 4C). GAD67 was highly expressed throughout the grafted cells, corroborating the GABAergic identity of the CR+ neurons (Figure 4C). Finally, we ruled out any unwanted effects of cyclosporine A on the differentiation and survival of the cells, by treating *in vitro* cultures for up to 7 weeks (Figure S3). These results show that hESC-derived MGE/CGE-like progenitors have the capacity to differentiate into cells resembling striatal interneurons *in vivo*, as well as adopting septal and hippocampal interneuron-like fates having settled in these regions.

DISCUSSION

In this study, we have shown that hESCs can differentiate into striatal interneurons. *In vitro*, MGE/CGE-like progenitors gave rise to SST+, PV+, and CR+ neurons, which normally populate the cortex, striatum, and hippocampus. After transplantation into the neonatal rat striatum, they produced striatal interneuron-like cells expressing CR and ChAT. Consistent with this, both *EPHB1/3* (striatal) and *NRP1* (cortical) transcripts were present in our cultures (Marin et al., 2001; Villar-Cervino et al., 2015). Thus, striatal transplantation of hESC-derived MGE or CGE progenitors results in a bias toward striatal interneuron fate, rather than cortical interneuron fate following cortical transplantation.

The discrepancies between interneuron subtypes obtained in culture and in the rat striatum are intriguing. Neuronal differentiation *in vivo* was delayed in comparison with *in vitro* cultures—a phenomenon we have observed previously in hESC-MSN transplantation (Arber et al., 2015). CR interneurons appear relatively early in human cortical development, around gestational week 6, whereas SST and PV interneurons appear only sparsely around gestational week 20, perhaps explaining their total absence from the grafted cells in this study (Maroof et al., 2013; Nicholas et al., 2013; Zecevic et al., 2011). In the human striatum, there are three times more CR interneurons than PV or SST (Wu and Parent, 2000). Studies in mice and humans have shown that not all CR interneurons are derived from the CGE, and that most striatal CR interneurons are MGE-derived (Marin et al., 2000; Wang et al., 2014). Furthermore, research has shown that the host brain region is able to alter the fate of transplanted cells to more closely resemble its own (Quattrocolo et al., 2017). It is therefore reasonable to hypothesize that local cues favored either the survival of fate-committed CR and ChAT neurons, or their differentiation from the surviving progenitors. Future work could be designed to address this question by transplanting MGE/CGE-like progenitors to different brain regions, or at a later time point when they might be more fate-committed.

Neurotherapeutic strategies for HD may require the inclusion of striatal interneurons, as proof-of-concept has been provided by transplanting WGE—containing both MGE and LGE—into rodents, monkeys, and human patients (Barker et al., 2013; Kendall et al., 1998; Lelos et al., 2016; Palfi et al., 1998). Experimental HD therapy using hESC-derived MSNs also suggests a potential role for interneurons (Ma et al., 2012; Wu et al., 2018). These hESC-MSN preparations likely contain interneurons as they are induced by SHH using paradigms similar to interneuron induction (Ma et al., 2012). Therefore, optimizing striatal interneuron differentiation, maintenance, and transplantation will be vital for future *in vitro* and *in vivo* studies into striatal development, function and repair.

This article presents an original application for hPSC-derived MGE/CGE-like interneurons. We have highlighted a need for better understanding of the mechanisms behind the fate determination of forebrain interneurons and their role within the striatum. The stark differences in striatal interneuron numbers between humans and rodents

Figure 3. Transplanted MGE/CGE-like Progenitors Adopt Region-Specific Morphologies

(A) Representative immunohistochemistry images of H7-tauGFP+ (green) hESC-derived progenitors and neurons stained for HuNu (red) and counterstained with DAPI (blue) at 6, 12, and 20 weeks post-transplantation, in the striatum, septum, and hippocampus of rats. Visible GFP+ cells were traced using Neurolucida and representative examples are shown on the right. Scale bar, 50 μm.

(B) Sholl profiles comparing cells in each brain region at 6, 12, and 20 weeks. Data presented are mean number of intersections per shell ± SEM of n = 9–26 cells.

(C) The number of primary neurites, branch points, and terminations were compared by two-way ANOVA with *post hoc* Bonferroni. Horizontal bars show significant differences across time points color-coded to their respective brain regions (blue, striatum; red, septum; green, hippocampus) and black vertical bars show significant differences between brain regions at 20 weeks. *$p < 0.05$, **$p < 0.01$, ***$p < 0.001$.

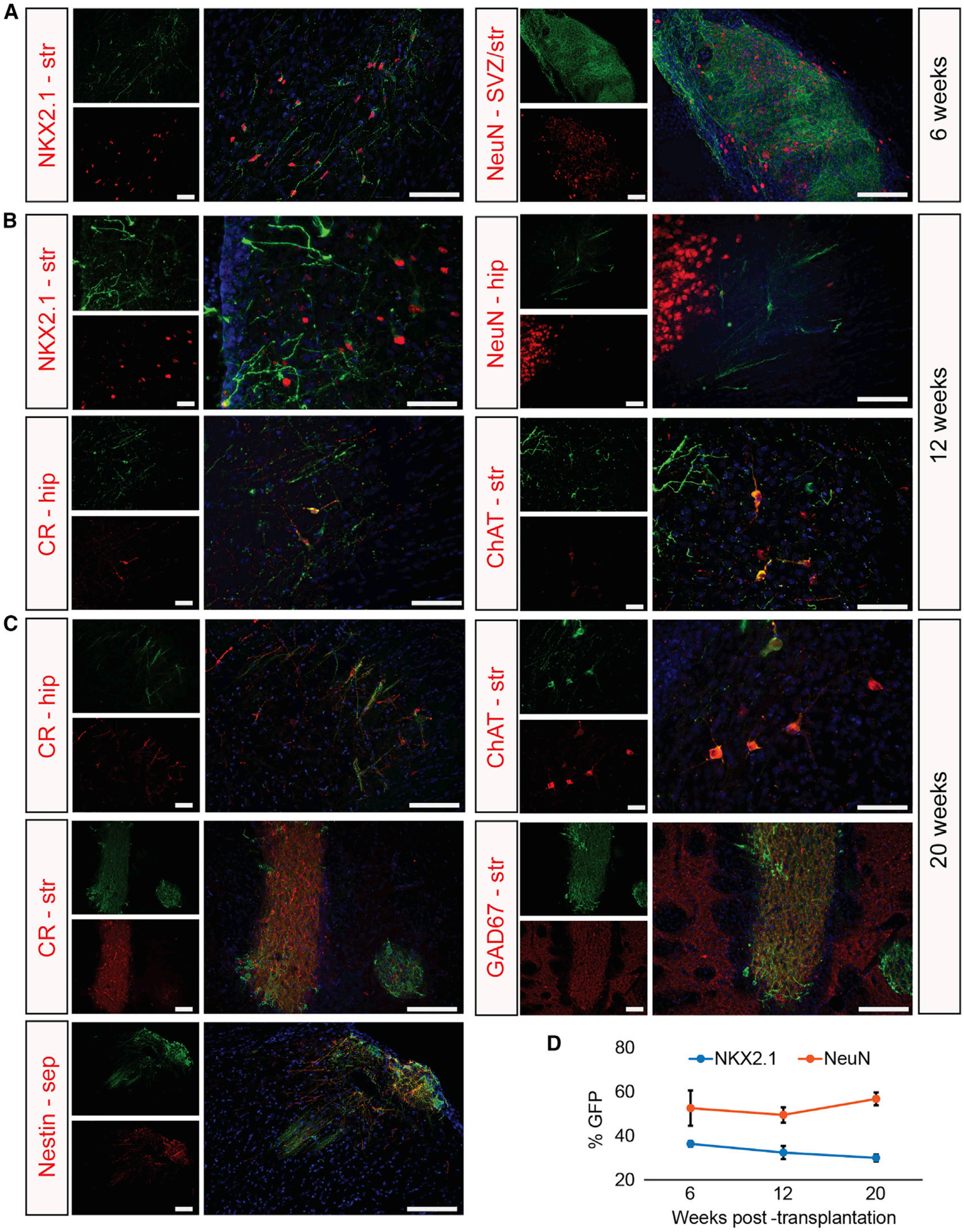

Figure 4. Transplanted MGE/CGE-like Progenitors Differentiate into Striatal Interneurons

(A–C) Representative immunohistochemistry images of brain sections from 6 (A), 12 (B), and 20 (C) weeks post-transplantation, with GFP$^+$ (green) transplanted cells and DAPI (blue).

(D) NKX2.1 (blue: 6 weeks, n = 6; 12 weeks, n = 5; 20 weeks, n = 3) and NeuN (orange: 6 weeks, n = 5; 12 weeks, n = 3; 20 weeks, n = 3) were counted as a percentage of HuNu$^+$ and GFP$^+$ cells, respectively. Two-way ANOVA reported no significant differences. Str, striatum; SVZ, subventricular zone; hip, hippocampus; sep, septum.

Scale bars, 100 μm (A–C, top two panels), 250 μm (C, bottom three panels).

indicates that we should be looking for such answers in human cells.

EXPERIMENTAL PROCEDURES

HESC Culture and Differentiation

Three independent hESC lines (H7, H9, and iCas9-HUES9) and an H7 derivative line (H7-tauGFP) were used in this study. Routine hESC culture and interneuron differentiation methods, and the derivation of the H7-tauGFP$^+$ cells are provided in Supplemental Experimental Procedures.

Transplantation

All animal work was done in compliance with the European Directive 2010/63/EU on the protection of animals used for scientific purposes. All surgical procedures and injection coordinates are described in Supplemental Experimental Procedures.

Statistical Analyses

All data were collected from at least three independent experiments, and are presented as mean ± SEM. Data were tested for normality with the Shapiro-Wilk test, and for equal variance with the Levene test, before performing statistical analyses by unpaired t test, ANOVA or non-parametric alternatives as stated in the figure legends. *Post hoc* Bonferroni test was applied following ANOVA to correct for multiple comparisons. All statistical tests were performed in Origin (OriginLab) or SPSS (IBM).

SUPPLEMENTAL INFORMATION

Supplemental Information includes Supplemental Experimental Procedures, three figures, and three tables and can be found with this article online at https://doi.org/10.1016/j.stemcr.2018.12.014.

AUTHOR CONTRIBUTIONS

In vitro hESC culture, differentiation, qPCR and immunostaining was carried out by Z.N., F.K., C.T., and M.C. Electrophysiology was performed by Z.N. *In vivo* work was conducted by Z.N. and C.M.K., and Z.N. did perfusions, tissue sectioning and immunohistochemistry. All imaging, cell counting and *post hoc* analyses were done by Z.N. or F.K. S.B.D., A.C.E., and M.L. provided guidance and conceptual support. All authors edited the manuscript.

ACKNOWLEDGMENTS

We thank Dr Niels Haan for kindly donating mouse astrocytes for the co-culture electrophysiology experiments and Dr Marija Fjodorova for her invaluable expertise. Thanks also to Dr Mariah Lelos and all members of the ML laboratory for helpful discussions during the course of the study. This work was supported by the UK Medical Research Council. A.C.E. is supported by a Jane Hodge Foundation Neuroscience Research Fellowship.

Received: June 8, 2018
Revised: December 17, 2018
Accepted: December 18, 2018
Published: January 17, 2019

REFERENCES

Arber, C., Precious, S.V., Cambray, S., Risner-Janiczek, J.R., Kelly, C., Noakes, Z., Fjodorova, M., Heuer, A., Ungless, M.A., Rodriguez, T.A., et al. (2015). Activin A directs striatal projection neuron differentiation of human pluripotent stem cells. Development *142*, 1375–1386.

Barker, R.A., Mason, S.L., Harrower, T.P., Swain, R.A., Ho, A.K., Sahakian, B.J., Mathur, R., Elneil, S., Thornton, S., Hurrelbrink, C., et al. (2013). The long-term safety and efficacy of bilateral transplantation of human fetal striatal tissue in patients with mild to moderate Huntington's disease. J. Neurol. Neurosurg. Psychiatry *84*, 657–665.

Bissonnette, C.J., Lyass, L., Bhattacharyya, B.J., Belmadani, A., Miller, R.J., and Kessler, J.A. (2011). The controlled generation of functional basal forebrain cholinergic neurons from human embryonic stem cells. Stem Cells *29*, 802–811.

Butt, S.J., Fuccillo, M., Nery, S., Noctor, S., Kriegstein, A., Corbin, J.G., and Fishell, G. (2005). The temporal and spatial origins of cortical interneurons predict their physiological subtype. Neuron *48*, 591–604.

Cambray, S., Arber, C., Little, G., Dougalis, A.G., de Paola, V., Ungless, M.A., Li, M., and Rodriguez, T.A. (2012). Activin induces cortical interneuron identity and differentiation in embryonic stem cell-derived telencephalic neural precursors. Nat. Commun. *3*, 841.

Capetian, P., Pauly, M.G., Azmitia, L.M., and Klein, C. (2014). Striatal cholinergic interneurons in isolated generalized dystonia-rationale and perspectives for stem cell-derived cellular models. Front. Cell. Neurosci. *8*, 205.

Crompton, L.A., Byrne, M.L., Taylor, H., Kerrigan, T.L., Bru-Mercier, G., Badger, J.L., Barbuti, P.A., Jo, J., Tyler, S.J., Allen, S.J., et al. (2013). Stepwise, non-adherent differentiation of human pluripotent stem cells to generate basal forebrain cholinergic neurons via hedgehog signaling. Stem Cell Res. *11*, 1206–1221.

Graveland, G.A., and DiFiglia, M. (1985). The frequency and distribution of medium-sized neurons with indented nuclei in the primate and rodent neostriatum. Brain Res. *327*, 307–311.

Kendall, A.L., Rayment, F.D., Torres, E.M., Baker, H.F., Ridley, R.M., and Dunnett, S.B. (1998). Functional integration of striatal allografts in a primate model of Huntington's disease. Nat. Med. *4*, 727–729.

Kim, T.G., Yao, R., Monnell, T., Cho, J.H., Vasudevan, A., Koh, A., Peeyush, K.T., Moon, M., Datta, D., Bolshakov, V.Y., et al. (2014). Efficient specification of interneurons from human pluripotent stem cells by dorsoventral and rostrocaudal modulation. Stem Cells *32*, 1789–1804.

Lelos, M.J., Roberton, V.H., Vinh, N.N., Harrison, C., Eriksen, P., Torres, E.M., Clinch, S.P., Rosser, A.E., and Dunnett, S.B. (2016). Direct comparison of rat- and human-derived ganglionic eminence tissue grafts on motor function. Cell Transplant. *25*, 665–675.

Lewis, D.A. (2012). Cortical circuit dysfunction and cognitive deficits in schizophrenia – implications for preemptive interventions. Eur. J. Neurosci. *35*, 1871–1878.

Ma, L., Hu, B., Liu, Y., Vermilyea, S.C., Liu, H., Gao, L., Sun, Y., Zhang, X., and Zhang, S.-C. (2012). Human embryonic stem cell-derived GABA neurons correct locomotion deficits in quinolinic acid-lesioned mice. Cell Stem Cell *10*, 455–464.

Marin, O., Anderson, S.A., and Rubenstein, J.L. (2000). Origin and molecular specification of striatal interneurons. J. Neurosci. *20*, 6063–6076.

Marin, O., Yaron, A., Bagri, A., Tessier-Lavigne, M., and Rubenstein, J.L. (2001). Sorting of striatal and cortical interneurons regulated by semaphorin-neuropilin interactions. Science *293*, 872–875.

Maroof, A.M., Brown, K., Shi, S.-H., Studer, L., and Anderson, S.A. (2010). Prospective isolation of cortical interneuron precursors from mouse embryonic stem cells. J. Neurosci. *30*, 4667–4675.

Maroof, A.M., Keros, S., Tyson, J.A., Ying, S.W., Ganat, Y.M., Merkle, F.T., Liu, B., Goulburn, A., Stanley, E.G., Elefanty, A.G., et al. (2013). Directed differentiation and functional maturation of cortical interneurons from human embryonic stem cells. Cell Stem Cell *12*, 559–572.

Nicholas, C.R., Chen, J., Tang, Y., Southwell, D.G., Chalmers, N., Vogt, D., Arnold, C.M., Chen, Y.J., Stanley, E.G., Elefanty, A.G., et al. (2013). Functional maturation of hPSC-derived forebrain interneurons requires an extended timeline and mimics human neural development. Cell Stem Cell *12*, 573–586.

Nobrega-Pereira, S., Kessaris, N., Du, T., Kimura, S., Anderson, S.A., and Marin, O. (2008). Postmitotic Nkx2-1 controls the migration of telencephalic interneurons by direct repression of guidance receptors. Neuron *59*, 733–745.

Palfi, S., Conde, F., Riche, D., Brouillet, E., Dautry, C., Mittoux, V., Chibois, A., Peschanski, M., and Hantraye, P. (1998). Fetal striatal allografts reverse cognitive deficits in a primate model of Huntington disease. Nat. Med. *4*, 963–966.

Powell, E.M., Campbell, D.B., Stanwood, G.D., Davis, D., Noebels, J.l., and Levitt, P. (2003). Genetic disruption of cortical interneuron development causes region- and GABA cell type-specific deficits, epilepsy, and behavioral dysfunction. J. Neurosci. *23*, 622–631.

Pratt, T., Sharp, L., Nichols, J., Price, D.J., and Mason, J.O. (2000). Embryonic stem cells and transgenic mice ubiquitously expressing a tau-tagged green fluorescent protein. Dev. Biol. *228*, 19–28.

Quattrocolo, G., Fishell, G., and Petros, T.J. (2017). Heterotopic transplantations reveal environmental influences on interneuron diversity and maturation. Cell Rep. *21*, 721–731.

Reiner, A., Shelby, E., Wang, H., Demarch, Z., Deng, Y., Guley, N.H., Hogg, V., Roxburgh, R., Tippett, L.J., Waldvogel, H.J., et al. (2013). Striatal parvalbuminergic neurons are lost in Huntington's disease: implications for dystonia. Mov. Disord. *28*, 1691–1699.

Villar-Cervino, V., Kappeler, C., Nobrega-Pereira, S., Henkemeyer, M., Rago, L., Nieto, M.A., and Marin, O. (2015). Molecular mechanisms controlling the migration of striatal interneurons. J. Neurosci. *35*, 8718–8729.

Wang, C., You, Y., Qi, D., Zhou, X., Wang, L., Wei, S., Zhang, Z., Huang, W., Liu, Z., Liu, F., et al. (2014). Human and monkey striatal interneurons are derived from the medial ganglionic eminence but not from the adult subventricular zone. J. Neurosci. *34*, 10906–10923.

Wu, M., Zhang, D., Bi, C., Mi, T., Zhu, W., Xia, L., Teng, Z., Hu, B., and Wu, Y. (2018). A chemical recipe for generation of clinical-grade striatal neurons from hESCs. Stem Cell Reports *11*, 635–650.

Wu, Y., and Parent, A. (2000). Striatal interneurons expressing calretinin, parvalbumin or NADPH-diaphorase: a comparative study in the rat, monkey and human. Brain Res. *863*, 182–191.

Zecevic, N., Hu, F., and Jakovcevski, I. (2011). Interneurons in the developing human neocortex. Dev. Neurobiol. *71*, 18–33.

Zikopoulos, B., and Barbas, H. (2013). Altered neural connectivity in excitatory and inhibitory cortical circuits in autism. Front. Hum. Neurosci. *7*, 609.

Stem Cell Reports, Volume *12*

Supplemental Information

Human Pluripotent Stem Cell-Derived Striatal Interneurons: Differentiation and Maturation *In Vitro* and in the Rat Brain

Zoe Noakes, Francesca Keefe, Claudia Tamburini, Claire M. Kelly, Maria Cruz Santos, Stephen B. Dunnett, Adam C. Errington, and Meng Li

Supplemental Figures

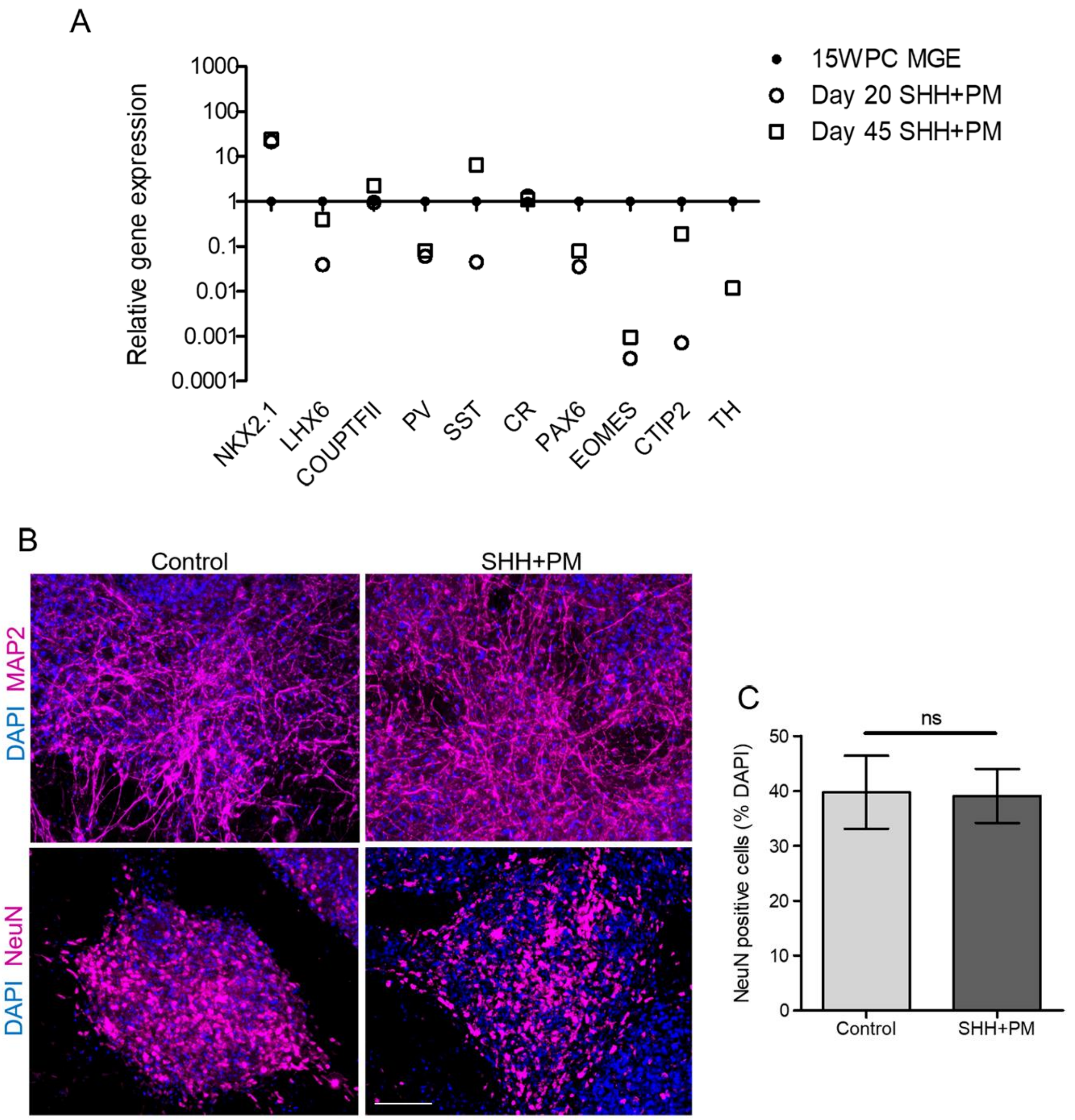

Figure S1. Additional characterisation of hESC-neural derivatives. (A) qPCR data in figure 1 presented as gene expression fold-change of SHH-treated relative to 15 weeks old human fetal MGE tissue. Data are presented as mean fold-change ± SEM from 3 independent replicates performed in a H7 subclone. Data on human fetal tissues was an average of two independent samples. (B) MAP2 and NeuN antibody staining of day 55 control and SHH+PM treated cultures, counterstained with DAPI (blue). A similar proportions of $MAP2^{+}$ and $NeuN^{+}$ cells were present in both conditions. (C) Counting data for NeuN staining. Graph shows mean +/- SEM. No significant difference between NeuN count between treatments ($p>0.05$ unpaired two-tailed t-test). Scale bar: 150μm.

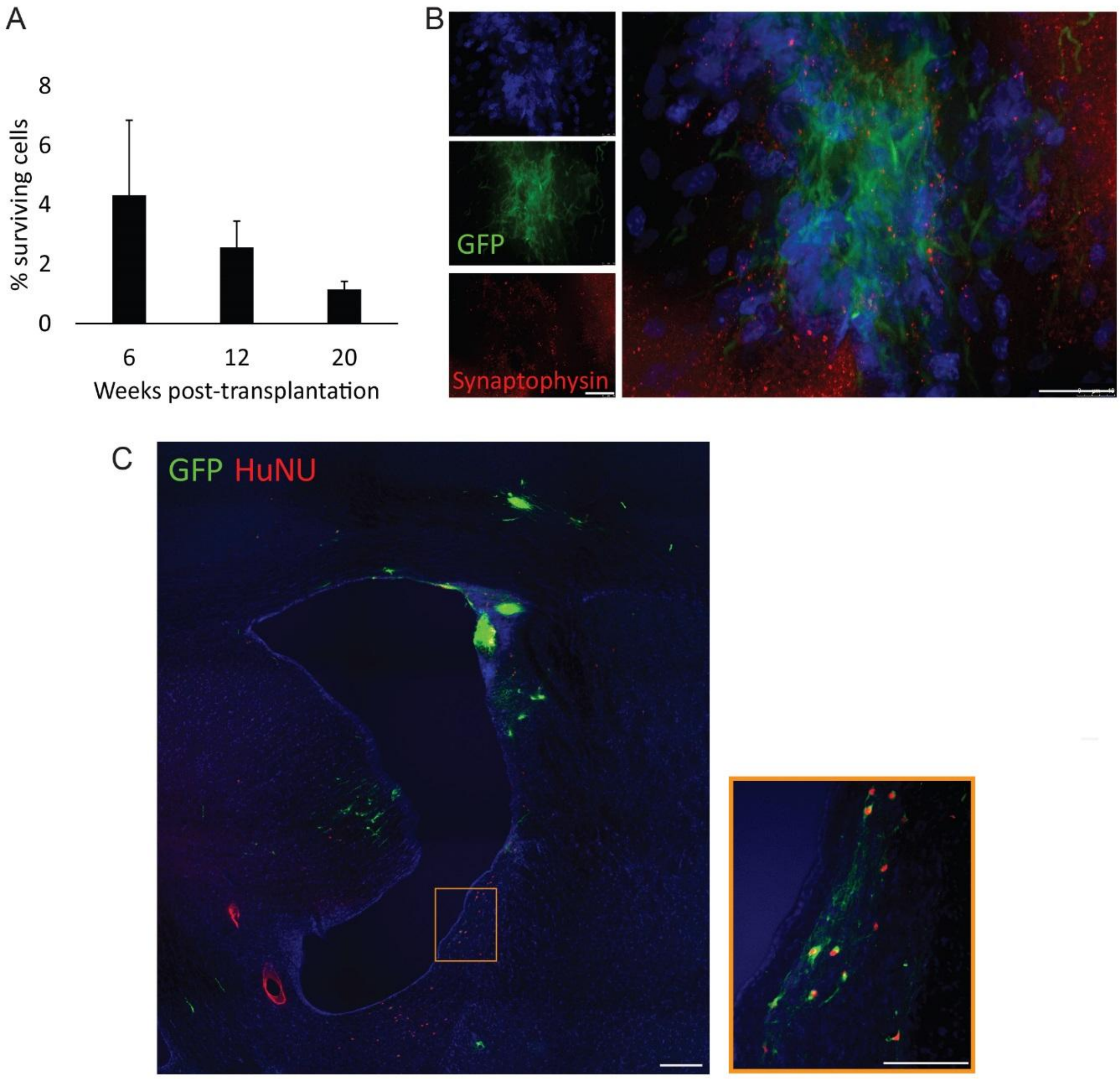

Figure S2. Migration and synapse formation of grafted human neurons

(A) Bar graph showing the mean ± SEM percentage of surviving cells (from 2 x 10^5 cells grafted) at each time point. (B) Immunocytochemistry image of synaptic marker Synaptophysin expressed within a clump of GFP+ grafted cells. (C) Tilescan immunocytochemistry image from 12 weeks post-transplantation showing potential migratory path of cells from striatum towards septum and hippocampus, with magnified yellow box to show HuNu/GFP co-expression. Scale bars: 100 μm.

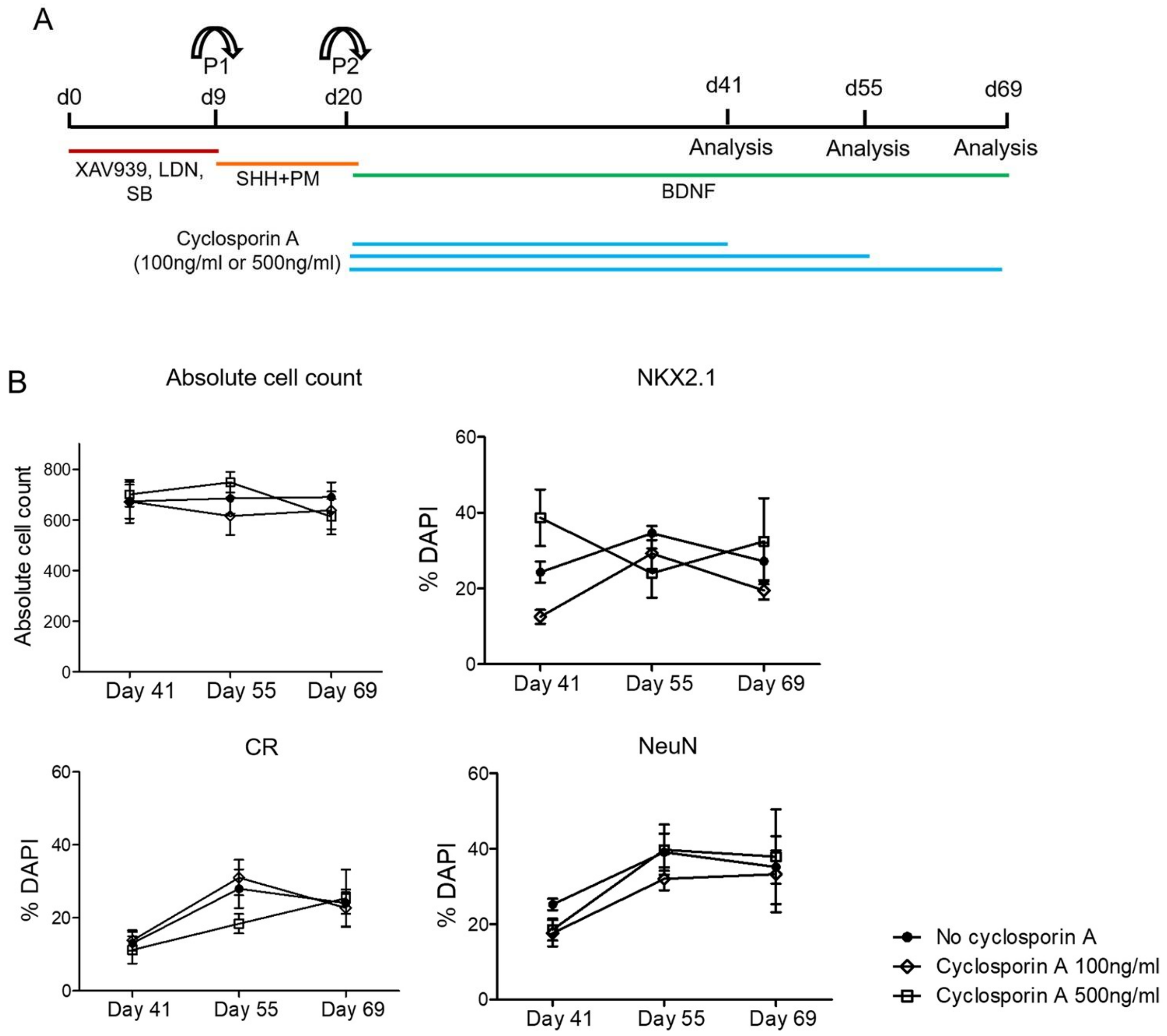

Figure S3. Cyclosporin A treatment had no effect on cell proliferation/survival or the cell fate. (A)Schematics of Cyclosporin A treatment. Cultures were exposed with or without cyclosporine A for 3 weeks (day 20 until day 41), 5 weeks (day 20 until day 55) and 7 weeks (day 20 until day 69) at either 100ng/ml or 500ng/ml cyclosporin A. Staining for each marker was carried out for all groups at indicated time point. No significant difference was found between cyclosporin A treated and control cultures (P>0.5, two-way ANOVA) for any of the markers. Data represent mean +/- SEM of three biological replicates for the markers indicated.

Supplemental tables

Table S1. qPCR data for Figures 1 and S1

Time point	qPCR target gene	Mean fold-change	SEM	P value
D20	*NKX2.1*	3427.60	270.45	0.003
	LHX6	21.45	5.74	0.003
	COUP-TFII	0.83	0.08	0.142
	PAX6	0.01	0.00	0.001
	EMX1	0.01	0.01	0.022
	EOMES	0.01	0.00	0.001
D45	*NKX2.1*	2729.88	0.62	0.003
	LHX6	241.65	0.15	0.001
	COUP-TFII	2.70	0.15	0.032
	PV	5.10	0.28	0.043
	SST	1.45	0.24	0.202
	CR	0.22	0.30	0.045
	PAX6	0.01	0.23	0.001
	EOMES	0.38	0.41	0.120
	CTIP2	0.20	0.28	0.033
	TH	0.57	0.35	0.832
	EphB1	2.38	0.19	0.051
	EphB3	2.26	0.22	0.070
	NRP1	2.99	0.20	0.034

Table S2. Immunocytochemistry data for Figures 1 and S1

Time point	ICC target protein	Control		SHH+PM		P value
		Mean	SEM	Mean	SEM	
D20	NKX2.1	3.0	2.1	72.3	3.5	0.000
	FOXG1	75.8	1.6	64.9	5.0	0.439
	ASCL1	0.1	0.0	23.0	0.6	0.018
	OLIG2	1.5	0.6	25.7	4.2	0.000
	COUP-TFII	22.2	3.9	22.0	4.7	0.169
D60	NKX2.1	7.2	2.2	42.1	5.1	0.000
	SST	1.3	0.5	6.3	1.3	0.000
	PV	0.0	0.0	1.5	0.9	0.116
	CR	3.3	0.8	3.9	0.4	0.620

Table S3. Electrophysiology data for Figure 2

Measurement	Unit	D45		D60		P value
		Mean	SEM	Mean	SEM	
Resting membrane potential	mV	-33.7	1.89	-39.3	1.84	0.022
Input resistance	MΩ	1394.6	153.4	1368.1	168.1	0.545
Membrane time constant	ms	81.0	8.13	98.4	10.14	0.189
Capacitance†	pF	63.7	4.04	81.3	10.81	0.072
Spontaneous event frequency	Hz	0.43	0.08	0.45	0.15	0.451
Evoked spike frequency	Hz	9.39	2.05	7.05	2.21	0.778
Spike amplitude	mV	63.3	3.33	67.1	3.61	0.221
Spike half-width	ms	5.9	0.42	3.8	0.28	0.000
Total neurite length	μm	1786.1	166.1	2573.0	283.9	0.015
Primary path length	μm	207.7	46.4	220.4	41.8	0.420
Branch length	μm	1578.4	178.2	2352.6	301.6	0.022

†Equal variance not assumed

Supplemental experimental procedures

HESC culture and differentiation

HESCs – H7 and derivative lines (H7-tauGFP), H9 or iCas9-HUES9 (González *et al.* 2014) – were cultured on hESC-qualified Matrigel and kept in TeSR-E8 medium. Neural induction was initiated as previously described in (Arber et al., 2015) with additional ventral patterning based on (Maroof et al., 2010; Maroof et al., 2013). Briefly, cells were grown to 90% confluency in TeSR-E8 before switching to (as day 0) N2B27 medium supplemented with SB431542 (10 µM, Tocris), LDN193189 (100 nM, Sigma) and XAV939 (2 µM, Tocris) from day 0-9. SHH (200 ng/ml, C24II R&D) and Purmorphamine (1 µM, Millipore) were added from day 10 to day 18 and BDNF (10 ng/ml, Peprotech) from day 25 onwards. Cultures were passaged on days 9 and 20 using 0.02% EDTA and seeded onto plates coated with fibronectin (15 µg/ml) or poly-D-lysine (10 µg/ml) and laminin (10 µg/ml), respectively.

Derivation of H7-GFP cell line

A mammalian expression vector CAG-tauGFP was used to generate a H7 derivative line constitutively expressing a tauGFP fusion protein(Pratt et al., 2000). The puromycin resistant gene (*pac*) is linked downstream of the *tauGFP* fusion gene via an internal ribosome entry site (IRES) to ensure that all puromycin resistan cells co-express tauGFP. Plasmid DNA was transfected into H7 hESCs using the Lonza 4D-Nucleofector as per manufacture instruction. 48 hours after nucleofection, cells were selected in the presence of 1µg/ml puromycin for 10 days. Surviving colonies were isolated and verified for stable GFP expression throughout neuronal differentiation. The best line (named H7-GFP) that exhibited high level and constitutive GFP expression in the derived neuronal cells was used for electrophysiology and transplantation experiments. The H7-GFP cells behaved similarly in interneuron differentiation compared to the H7 parental line and other two hESC lines tested.

Electrophysiology

Primary mouse astrocytes were isolated from the cortices of postnatal day 7 C57BL/6 mouse pups and then seeded onto PDL-laminin-coated 13 mm glass coverslips. GFP-expressing neural progenitors were dissociated using Accutase (Thermo Fisher) on differentiation day 20 and seeded on top of the astrocytes at a density of 80,000 cells per cm^2. Cells were initially grown in a specialised culture

medium (BrainPhys) designed to aid functional maturation (Bardy et al., 2015) supplemented with a cocktail of growth factors and small molecules for 7 days followed by maintenance in BrainPhys medium alone (Telezhkin et al., 2016).

For whole cell patch clamp experiments, coverslips were transferred to a recording chamber maintained at room temperature (20-21°C) on the stage of an Olympus BX61W (Olympus) differential interference contrast (DIC) microscope and perfused at 2.5 ml/min with aCSF containing (in mM): 135 NaCl, 5 KCl, 1.2 MgCl2, 1.25 CaCl2, 10 D-glucose, 5 HEPES (Sigma), pH 7.4. GFP-expressing neurons were identified for recording by their fluorescence emission, which was viewed using a Rolera Bolt CMOS video rate camera (QImaging) following excitation by a 473 nm blue LED (CoolLED). Whole cell patch clamp recordings were performed using a MultiClamp 700B amplifier and pipettes with resistances of 4-8 MΩ when filled with an intracellular recording solution containing (in mM): 117 KCl (KGluc for voltage clamp), 10 NaCl, 11 HEPES, 2 Na2-ATP, 2 Na-GTP, 1.2 Na2-phosphocreatine, 2 MgCl2, 1 CaCl2 and 11 EGTA (Sigma), pH 7.2, supplemented with 0.1 AlexaFluor Hydrazide 555 (Thermo Fisher Scientific). Electrophysiological data were sampled at 20 kHz and filtered at 3 kHz using a Digidata 1550 analogue to digital converter and pClamp 10 software (Molecular Devices, USA). Series resistance was compensated using the bridge-balance and varied <20% during recordings. Recordings were not corrected for liquid junction potentials.

Resting membrane potential (RMP), input resistance (R_N), membrane time constant (τ) and membrane capacitance (C_m) were measured in current clamp mode. RMP was measured using the mean baseline membrane potential over a 10 second sampling period immediately after establishing whole-cell access. R_N was calculated using Ohm's law from the amplitude of the steady-state voltage deflection in response to a 1 second hyperpolarizing current injection (-10 pA). C_m was estimated according to $Cm = τ_m/R_N$ where $τ_m$ was calculated by fitting a single exponential function to the mean voltage response evoked by a series of hyperpolarizing current pulses (-10 pA, 1 s). For evoked activity measurement, cells were injected with current steps of 20 pA between -100 and +200 pA (1 s) to measure voltage responses. The properties of single action potentials were calculated from the first spike evoked by a rheobase current injection step.

Neurotransmitter-evoked currents were measured using voltage clamp recordings. Brief pulses (30 ms) of glutamate or GABA (100 µM) were applied using a custom built pressure application system via a second pipette positioned close to the soma of the recorded neuron. The mean neurotransmitter-evoked current was calculated as the average of 5 pressure applications delivered at 30 s intervals.

For morphological analysis, neurons were filled with Alexa 555 during whole cell recordings and their soma and complete dendritic tree imaged using a Rolera Bolt CMOS video rate camera (QImaging). Acquired fluorescence images were manually traced post-hoc using Neurolucida 360 (MBF biosciences, USA) and analysed using Neurolucida Explorer.

Data were analysed using Clampfit 10 software (Molecular Devices) and then exported to and plotted using Origin 9 (OriginLab).

Quantitative real-time PCR (qPCR)

Total RNA was extracted using Tri reagent treated with TURBO DNase. cDNA was generated using qScript cDNA synthesis kit. qPCR was performed with Mesa Green qPCR master mix with specific primers listed in table below and dissociation curves were recorded to check for amplification specificity. Cq values were normalised to two housekeeping reference genes and changes in expression calculated using the 2-∆∆CT method. Data shown are mean±SEM of three biological replicates performed in H7 cells and each sample was measured in triplicate on a CFX Connect Real Time PCR machine.

Immunocytochemistry

Cultured cells were washed with PBS and fixed in 3.7% PFA for 15 minutes at room temperature. For nuclear stains, fixed cells underwent 5 minute methanol washes of ascending then descending methanol dilutions in PBS (33% and 66% at room temperature, then 100% at -20°C). Cells were blocked for 30 minutes in PBS-T (0.3% Triton-X-100) with 2% BSA and 5% donkey serum. Primary antibodies were added in PBS-T with 3% donkey serum and left overnight at 4°C. Secondary antibodies (AlexaFluor anti-donkey 488, 555, 647; Life Technologies) were added in PBS-T and left for 1 hour at room temperature. DAPI (Molecular Probes) was used to counterstain cell nuclei. Staining was preserved using DAKO fluorescent mounting medium (Life Technologies). Samples were imaged on a Leica DMI6000b

fluorescent microscope and cells were counted manually with ImageJ (cytoplasmic markers) or using automated Cell Profiler (nuclear markers). Cell counts from at least 3 independent experiments were used for statistical analysis.

Immunohistochemistry

Coronal sections were cut on a freezing microtome to 40 µm thickness in a 1:12 series. Floating sections were blocked in Triton-X-100-TBS with 3% donkey serum for 1 hour, then incubated in TXTBS with 1% donkey serum and primary antibodies overnight. Sections were washed 3 times in TBS then incubated in TBS with secondary antibodies (1/200, AlexaFluor 488, 555, 594 and 647) overnight. After 3 more TBS washes sections were mounted onto glass slides, air dried, cover-slipped with VectaShield hardset antifade mounting medium with DAPI (Vector Labs) and stored at 4°C. Slides were imaged using a Leica DM6000B microscope. Neurolucida 360 software (MBF Bioscience) was used to manually reconstruct imaged neurons and conduct Sholl analysis and path length analysis.

Transplantation

All animal work was done in compliance with the European Directive 2010/63/EU on the protection of animals used for scientific purposes. All surgical procedures were carried out under isoflurane anaesthesia using a neonatal adaptor on a stereotaxic frame. Postnatal day 2 Sprague Dawley pups (n=15) were injected with 2×10^5 cells in a volume of 1 µl using a Hamilton syringe. Injection coordinates targeted the right striatum (0.9 mm anterior and 1.8 mm lateral to bregma, 2.0 mm below the dura; 0.7 mm anterior and 1.9 mm lateral to bregma, 2.9 mm below the dura). After immune rejection was observed in a pilot study, animals were given daily intraperitoneal Cyclosporine A injections (10 mg/kg; Sandimmun) from weaning onwards, and were sacrificed at 6, 12 and 20 weeks (n=6, 5 and 4) post-surgery by transcardial perfusion with 4% PFA and post-fixed for a further 4 hours.

List of qPCR primers

Gene	Forward primer	Reverse primer
GAPDH	ATGACATCAAGAAGGTGGTG	CATACCAGGAAATGAGCTTG
β-ACTIN	TCACCACCACGGCCGAGCG	TCTCCTTCTGCATCCTGTCG
NKX2.1	CGCATCCAATCTCAAGGAAT	TGTGCCCAGAGTGAAGTTTG
LHX6	GACGACATCCACTACACCCC	GGCCCATCCATATCGGCTTT
COUPTFII	GGAGAAGCTCAAGGCACTGCA	CCTGCAAGCTTTCCACATGGG
PAX6	AATAACCTGCCTATGCAACCC	AACTTGAACTGGAACTGACACAC
EMX1	ACCGGAGGACAAAGTACAAAC	TAGTCATTGGAGGTGACATCG
EOMES	CTGCCTACCAAAACACCGATATTAC	AGCGGGCTTGAGGTAAAGTG
PVALB	AAAGAGTGCGGATGATGTGAAG	ACCCCAATTTTGCCGTCCC
SST	GCTGCTGTCTGAACCCAAC	CGTTCTCGGGGTGCCATAG
CR	TCAGAGATGTCCCGACTCCTG	GCCGCTTCTATCCTTGTCGTAA
CTIP2	CTCCGAGCTCAGGAAAGTGTC	TCATCTTTACCTGCAATGTTCTCC
TH	GAGTACACCGCCGAGGAGATTG	GCGGATATACTGGGTGCACTGG
EPHB1	GCACATCTCTGGTGATTGCTC	ACGCTGTTCTCAGGCTCATAG
EPHB3	GGCCATAGCCTATCGGAAGT	TCCCAGTAGGGTCGCTCTC
NRP1	AAGGTTTCTCAGCAAACTACAGTG	GGGAAGAAGCTGTGATCTGGTC

List of primary antibodies

Antigen	Species	Supplier	Catalogue number	Dilution	
				Cells	Rat tissue
Calretinin	rabbit	Swant	7697	1/500	1/1000
ChAT	goat	Millipore	AB144	1/100	1/200
FOXG1	rabbit	Abcam	18259	1/250	
GAD67	mouse	Millipore	mab5406	1/500	1/500
GFP	rabbit	Invitrogen		1/500	1/1000
HuNu	mouse	Millipore	mab1281	1/250	1/1000
ASCL1	mouse	BD	556604	1/500	
Nestin	mouse	Neuromics		1/300	1/300
NeuN	mouse	Millipore	mab377	1/250	1/500
NKX2.1 (TTF1)	rabbit	Abcam	ab40880	1/1000	1/1000
NPY	rabbit	Immunostar	22940	1/200	1/250
Oct4	goat	Santa Cruz	sc8628	1/500	
OLIG2	goat	R&D		1/200	
Parvalbumin	mouse	Sigma	p3088	1/100	1/100
Somatostatin	rabbit	Millipore		1/50	
Somatostatin	rat	Millipore		1/50	1/100

References

Arber, C., Precious, S.V., Cambray, S., Risner-Janiczek, J.R., Kelly, C., Noakes, Z., Fjodorova, M., Heuer, A., Ungless, M.A., Rodriguez, T.A.*, et al.* (2015). Activin A directs striatal projection neuron differentiation of human pluripotent stem cells. Development *142*, 1375-1386.

Bardy, C., van den Hurk, M., Eames, T., Marchand, C., Hernandez, R.V., Kellogg, M., Gorris, M., Galet, B., Palomares, V., Brown, J.*, et al.* (2015). Neuronal medium that supports basic synaptic functions and activity of human neurons in vitro. Proceedings of the National Academy of Sciences of the United States of America *112*, E2725-2734.

Maroof, A.M., Brown, K., Shi, S.-H., Studer, L., and Anderson, S.A. (2010). Prospective Isolation of Cortical Interneuron Precursors from Mouse Embryonic Stem Cells. Journal of Neuroscience *30*, 4667-4675.

Maroof, A.M., Keros, S., Tyson, J.A., Ying, S.W., Ganat, Y.M., Merkle, F.T., Liu, B., Goulburn, A., Stanley, E.G., Elefanty, A.G.*, et al.* (2013). Directed differentiation and functional maturation of cortical interneurons from human embryonic stem cells. Cell stem cell *12*, 559-572.

Pratt, T., Sharp, L., Nichols, J., Price, D.J., and Mason, J.O. (2000). Embryonic stem cells and transgenic mice ubiquitously expressing a tau- tagged green fluorescent protein. Developmental biology *228*, 19-28.

Telezhkin, V., Schnell, C., Yarova, P., Yung, S., Cope, E., Hughes, A., Thompson, B.A., Sanders, P., Geater, C., Hancock, J.M.*, et al.* (2016). Forced cell cycle exit and modulation of GABAA, CREB, and GSK3beta signaling promote functional maturation of induced pluripotent stem cell-derived neurons. American journal of physiology. Cell physiology *310*, C520-541.
